# Quantitative blood flow velocity imaging using laser speckle flowmetry

**DOI:** 10.1038/srep25258

**Published:** 2016-04-29

**Authors:** Annemarie Nadort, Koen Kalkman, Ton G. van Leeuwen, Dirk J. Faber

**Affiliations:** 1 Department of Biomedical Engineering and Physics, Academic Medical Center, University of Amsterdam, P.O. Box 22700, 1100 DE Amsterdam, The Netherlands; 2 ARC Centre of Excellence for Nanoscale BioPhotonics, Macquarie University, Sydney 2109, NSW Australia

## Abstract

Laser speckle flowmetry suffers from a debated quantification of the inverse relation between decorrelation time (*τ*_*c*_) and blood flow velocity (*V*), i.e. 1/*τ*_*c*_ = α*V*. Using a modified microcirculation imager (integrated sidestream dark field - laser speckle contrast imaging [SDF-LSCI]), we experimentally investigate on the influence of the optical properties of scatterers on *α in vitro* and *in vivo*. We found a good agreement to theoretical predictions within certain limits for scatterer size and multiple scattering. We present a practical model-based scaling factor to correct for multiple scattering in microcirculatory vessels. Our results show that SDF-LSCI offers a quantitative measure of flow velocity in addition to vessel morphology, enabling the quantification of the clinically relevant blood flow, velocity and tissue perfusion.

Quantitative imaging of microcirculatory blood supply to tissues is of paramount importance for diagnosis, therapy planning and monitoring, e.g. of metabolic, vascular and critical diseases^1^,^2^. Quantifying cerebral blood flow gives insight into brain metabolism^3^, while visualizing angiogenic vasculature can aid in localizing tumours^4^,^5^ and monitoring their development and oxygen metabolism^6^,^7^,^8^. Important clinical microcirculation parameters are blood flow (blood volume per unit time in the vasculature) and perfusion (blood volume per volume tissue, per unit time). Both are intimately related to blood flow velocity *V* through the morphology of the vasculature (e.g. vessel diameters and vessel density).

Laser speckle flowmetry^9^ is a widely available vascular imaging tool with potential use at the bedside or during operations^10^,^11^,^12^,^13^,^14^,^15^,^16^,^17^,^18^. It provides a measure for blood flow velocity by quantifying the decrease in speckle contrast as a result of ‘blurring’ of dynamic speckles within a finite camera exposure time^9^,^19^. When red blood cells (RBCs) move, the speckle contrast *K*, defined as the ratio of the standard deviation (*σ*_*i*_) to the mean (<*I*>) of the pixel intensity, decreases with increasing ratio of the camera exposure time *T* and the characteristic timescale of the speckle dynamics *τ*_*c*_^20^,^21^.

The interpretation of *τ*_*c*_ as a measure of blood flow, velocity or tissue perfusion is confounded^22^,^23^,^24^, but represents a clinically relevant question. Albeit that the relation is verified to be of the form 1/*τ*_*c*_ = α*V* in flow phantom experiments, the exact value of the proportionality constant *α* is debated and has not been verified *in vivo*^24^,^25^. Suggestions for *α* ranged from a dependence on system parameters^24^,^26^,^27^ to a dependence on the optical properties of the scatterers^28^ and dependence on multiple scattering^24^,^28^. These predictions differ 1–2 orders of magnitude^25^. However, to become a true quantitative tool the assessment of *α* beyond speculation is a necessity.

Thereto, we integrated sidestream dark field (SDF) microscopy^29^,^30^ with laser speckle contrast imaging (LSCI)^21^. SDF-LSCI enables simultaneous, independent measurement of *τ*_*c*_ and *V* in microcirculatory vessels or phantom flow channels. Our recent study showed that correction for additional decorrelation sources (e.g. muscle movement, dynamic scattering outside the focal volume) improves the relation between *τ*_*c*_ and *V*. Since α was suggested to depend on scatterer size through the scatterer phase function^28^ and multiple scattering through the number of dynamic scattering events^24^,^28^, we designed an *in vitro* flow phantom to investigate on the influence of scatterer size and volume fraction. We place our results in theoretical context taking into account the optical properties and experimental geometry, resulting in a practical relation 1/*τ*_*c*_ = α_1_*A*(*N*) × *V* where α_1_ is the proportionality constant for single scattering and *A*(*N*) scales for the average number of dynamic scattering events *N*. Applying this theoretical framework to chick embryo and human microcirculation beds demonstrates the feasibility of LSCI for quantitative mapping of blood flow velocities *in vivo*. Importantly, LSCI additionally provides means to derive the clinically relevant parameters blood flow and perfusion from the vessel geometry in microcirculation images.

## Practical Approach

**Figure 1:**
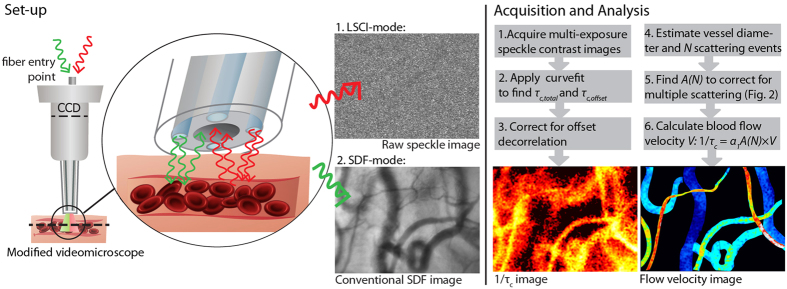
Overview of acquisition and analysis steps for quantitative laser speckle flowmetry. A modified SDF-LSCI videomicroscope enables consecutive multi-exposure laser speckle imaging and SDF-imaging of the same microcirculation area. The acquisition and analysis steps are supported by our theoretical modelling and experimental validation, as described in this Article.

Acquiring blood flow velocities and decorrelation times *in vivo* was realized using the integrated SDF-LSCI system as schematically shown in Fig. 1, enabling recording of raw speckle images and conventional SDF images of the same microcirculation area sequentially (see also Supplementary Fig. 1). To obtain quantitative flow velocity mapping several acquisition and analysis steps need to be taken. Step 1–3 describe the multi-exposure acquisition algorithm and its curve-fitting to find *τ*_*c*_^22^,^31^, where *τ*_*c,total*_ and *τ*_*c,offset*_ refer to *τ*_*c*_from vessel and tissue regions, respectively, and are required for our offset correction^21^. Step 4–6 describe the multiple scattering and scatterer phase function model to obtain the blood flow velocity, as elucidated in this Article. A more detailed description can be found in Supplementary Fig. 2, which, together with Fig. 1, serves as an overview of the outcomes in the rest of this paper and should be treated as a guideline to the reader.

## Theoretical Framework

When coherent light backscattered from different positions in biological tissue is imaged on a camera, the ensemble of phase differences gives rise to a randomly varying spatial intensity distribution (speckle). Movement of scatterers results in a temporally fluctuating speckle pattern. The characteristic timescale *τ*_*c*_ of the fluctuation can be used to quantify the motion of scattering particles. The parameter *τ*_*c*_ parameterizes the temporal electric field autocorrelation function (ACF) *g*_*1*_(τ) describing the sample dynamics. However, *g*_*1*_(τ) and thus *τ*_*c*_ depend on optical and physical properties of the tissue, e.g. the scattering phase function. Moreover, scattering from more than one moving particle causes faster decorrelation of the speckle pattern (shorter *τ*_*c*_). We review the optical properties relevant to our application and their relationship with *g*_*1*_(τ) and *τ*_*c*_ in Supplementary Section IIIa and IIIb, respectively. Our approach builds upon the theoretical foundation of Laser Doppler Flowmetry (LDF)^28^,^32^, Diffusing Wave Spectroscopy (DWS)^33^,^34^ and using our recent advances in the modelling of optical scattering of whole blood using Mie-Percus-Yevick (MPY) equations^35^. For high volume fractions of scatterers, as in blood, inter-particle correlation (“dependent scattering”) has to be taken into account to calculate the scattering properties (e.g. non-linear increase of the scattering coefficient with volume fraction). This correlation is implemented by modelling flowing blood as a discrete random medium of hard spheres which is characterized by average density and pair correlation function. The latter is calculated using Percus-Yevick (PY) equations^36^,^37^ as before^35^,^38^. Next the number of dynamic scattering events has to be determined. The probability density function for the number of scattering events per unit volume *p*_*N*_(*n*) is generally taken to be Poissonian^28^. Since this is only valid for low volume fractions, for RBCs in whole blood (volume fraction or hematocrit (Hct) 30–50%) we use a Gaussian distribution for *p*_*N*_ (*n*), where the relation between mean (*N*) and variance (*σ*_*n*_^2^) is determined by the pair correlation function^39^,^40^ (Supplementary Section IIIc). Here, *N* is estimated from Monte Carlo simulations of our measurement geometry (Supplementary Section IV). The result is a model of *g*_*1*_(τ) and associated *τ*_*c*_ in terms of optical properties, organization and scattering order.

**Figure 2:**
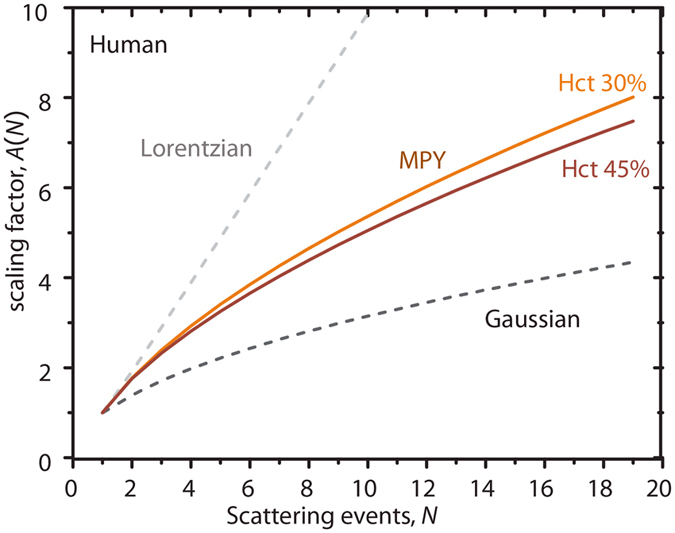
Multiple scattering scaling factor. Scaling factor *Α*(*N*) = α/α_*1*_ = *τ*_*c,1*_/*τ*_*c*_, is calculated using our model for autocorrelation *g*_*1*_(τ) based on Mie-Percus-Yevick scattering in human blood for Hct = 30% (upper solid orange line) and Hct = 45% (lower solid red line). As a practical guideline, *A*(*N*) from our MPY model can approximately be fitted by *A*(*N*) = 1.1 *N*^(2/3)^, which gives an error <10% for the range plotted for both hematocrits. Dashed lines represent *Α*(*N*) for Lorentzian (grey) and Gaussian (black) models for *g*_*1*_(τ). All scaling factors are calculated using a normal distribution for the number of scattering events in the vessel *p*_*N*_(*n*), with mean *N* and variance determined by the Percus-Yevick pair correlation function (Supplementary equation 6).

To fulfil our aim to quantify the relationship 1/*τ*_*c*_ = α*V* in multiple scattering scenarios, we introduce the rescale parameter *A*(*N*), defined as *A*(*N*) = *τ*_*c,1*_/*τ*_*c*_ or *A*(*N*) = α/α_*1*_, where the subscript ‘1’ denotes single scattering. Using the theoretical model outlined above we calculated *A*(*N*) for RBCs and a range of values for *N*, as plotted in Fig. 2, for two hematocrit values corresponding to whole blood (Hct = 45%) and the microcirculation (Hct = 30%)^41^ (solid lines). For comparison to conventional LSCI approaches we also plotted *Α*(*N*) for Gaussian and Lorentzian shape of *g*_*1*,_(*τ*) (Fig. 2, dashed lines, see also the Discussion). The calculation of *Α*(*N*) practically relates *V* to *τ*_*c*_*in vivo* via 1/*τ*_*c*_ = α_*1*_*Α*(*N*) × *V*.

The characteristic timescale *τ*_*c*_ of temporal speckle dynamics can be derived from spatial intensity statistics of an imaged speckle pattern (Supplementary Section IIId). After the initial derivation^9^ essential improvements to the analysis were included^42^,^43^, for example accounting for a static component in the speckle pattern. When a Gaussian form of *g*_*1*_(τ) is appropriate^42^ – confirmed by our theoretical analysis – an analytical expression for speckle contrast *K*(*T/*τ_*c*_) can be derived for curve fitting, where *T* is the camera exposure time varied in the measurement. Next to the sought τ_*c*_ the model incorporates the parameters *β*_*M*_^21^,^44^,^45^ (the measurement geometric calibration constant) and *ρ* (the fraction dynamically scattered light where *ρ* = *I*_*f*_ /(*I*_*f*_ + *I*_*s*_), with *I*_*f*_ the detected intensity of the fluctuating scattered light and *I*_*s*_ the detected intensity of the light scattered by static components) that can both be measured. The resulting expression is Supplementary equation (12), from which *τ*_*c*_ can be reliably estimated by applying a multi-exposure acquisition scheme and subsequent nonlinear curve fit^16^,^22^,^31^.

## Influence of Size and Volume Fraction of Scatterers

**Figure 3:**
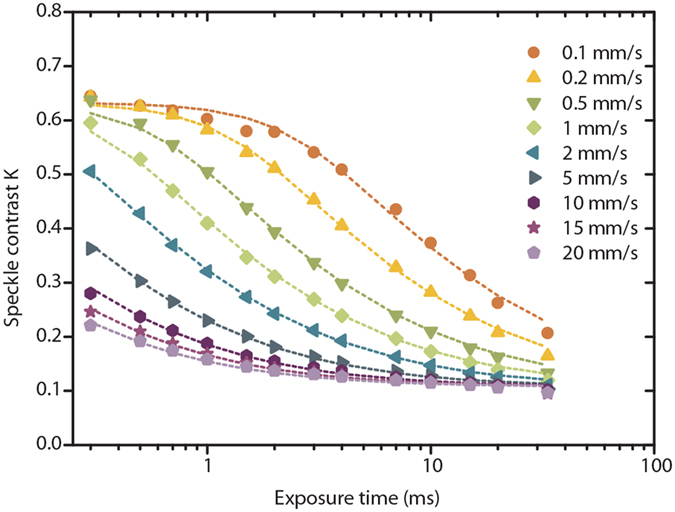
Multi-exposure curves and fits. Multi-exposure speckle contrast values (data points) and corresponding fit of Supplementary equation (12) (dashed lines) for 9 different flow velocities for 2 μm polystyrene spheres (2.5 vol%). Speckle contrast *K* is calculated according to Supplementary equation 12.

**Figure 4:**
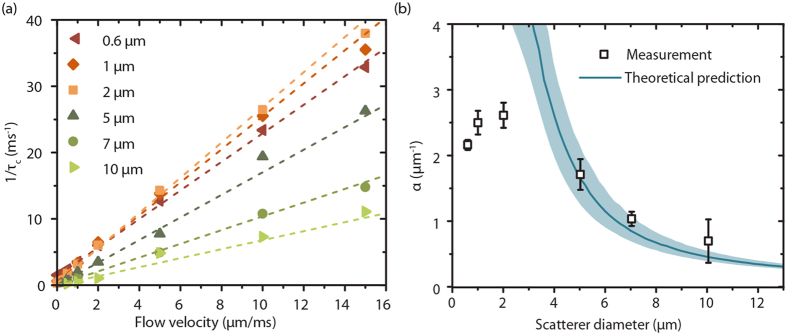
Influence of scatterer size on *α*. (**a)** 1/*τ*_*c*_ plotted against *V* for 6 different scatterer sizes (polystyrene microspheres diameter = [0.6–10 μm]), together with a linear fit (dotted lines) to the data points with weights *τ*_*c*_. The slope of the linear fit is *α*. No error bars are plotted for clarity, the average standard error on *τ*_*c*_ was 4% ± 2% (max. error 12%). **(b)** *α* versus scatterer diameter, error bars are 95% CI intervals from linear fit in (**a**). Also plotted is the theoretically derived *α* (solid line) using Mie-Percus-Yevick scattering theory and the number of scattering events *N* in the flow tube (diameter *d* 0.2 ± 0.03 mm) as obtained from Monte Carlo simulations (*N* = 1.2*μ*_*s*_*d*), *N* ranged from [2–15] for [0.6–10 μm] spheres. The shaded area represents the uncertainty in *α* due to error margins in optical properties of the scatterers.

Experimental LSCI data was acquired using an integrated SDF-LSCI device (Supplementary Fig. 1). Multi-exposure SDF-LSCI frames were recorded for 9 flow velocities [0.1–20 mm/s] and 6 differently sized microspheres [diameter 0.6–10 μm; all 2.5 vol%] and *τ*_*c*_ was estimated by fitting Supplementary equation (12) to the measured speckle contrast (see Methods). Typical multi-exposure curves and fits are shown in Fig. 3. In Fig. 4a, 1/τ_c_ vs. *V* is plotted for the different sphere sizes, where the slope defines *α*. For large scatterers (5, 7 and 10 μm) *α* increases with decreasing size. For small scatterers (0.6, 1 and 2 μm) however, *α* is approximately constant. The theoretical predictions for *α* follow from Supplementary Section III using the optical and physical properties listed in Supplementary Table 2 and Monte Carlo simulations for this geometry. Fig. 4b shows *α* plotted (data points) for all scatterer sizes, together with predictions (solid line). The shaded area represents the uncertainty in the calculation due to variations in size and refractive index of the scatterers.

**Figure 5:**
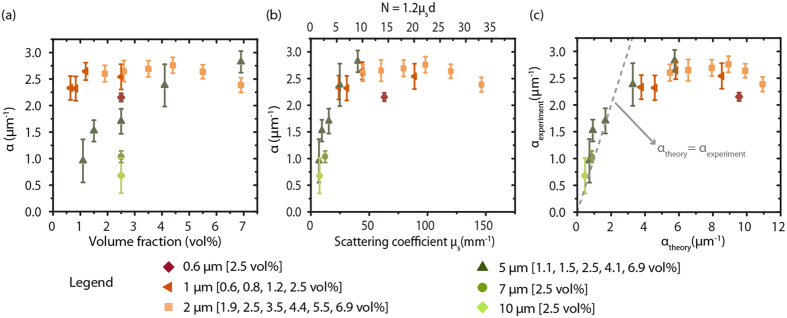
Influence of scatterer volume fraction on *α*. Measured *α* versus **(a)** scatterer volume fraction, **(b)** scattering properties (scattering coefficient *μ*_*s*_ and average number of scattering events *N* in the flow tube with diameter *d* 0.2 ± 0.03 mm, *N* = 1.2*μ*_*s*_*d*) and **(c)** theoretically derived *α* for all measured samples. Error bars are 95% CI intervals from linear fit on 1/*τ*_*c*_ vs. *V*.

In order to determine the effect of concentration, we prepared different volume fractions of solutions of 1, 2 and 5 μm spheres (Supplementary Table 2) and determined *α* shown in Fig. 5a together with *α* from 2.5 vol% solutions of 0.6, 7 and 10 μm. In Fig. 5b the same data points are plotted as a function of optical properties, scattering coefficient *μ*_*s*_ and *N*, which results in a more uniform increase of *α* with increasing *μ*_*s*_ resp. *N*, until *α* remains constant. Figure 5c shows measured values vs. prediction yielding good correspondence for small *α* (low *μ*_*s*_ and *N*), however for higher *α* theory overestimates the experimental measurement. We note that for *in vivo* blood flow velocity measurements small values for α are predicted.

## Quantitative Flowmetry *in vivo*

**Figure 6:**
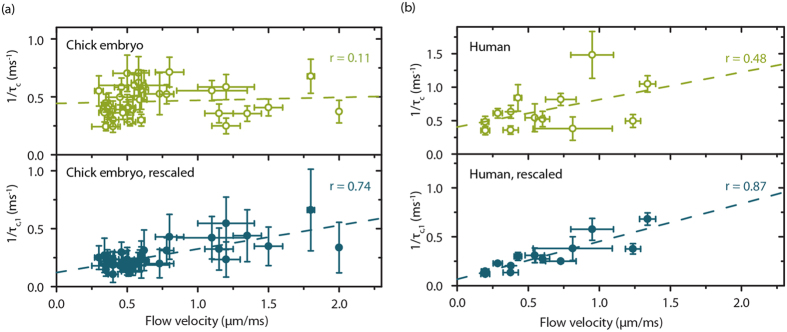
*In vivo* determination of *α*. 1/*τ*_*c*_ versus *V* for RBCs *in vivo*, for **(a)** chick embryo and **(b)** human microcirculation. The top panels (green open circles) show 1/τ_c_ estimated by a multi-exposure curve fit (Supplementary Section IIId, equation 12) and *τ*_*c,offset*_ correction (Supplementary equation (13)^21^). The bottom panels show 1/*τ*_*c,1*_ rescaled for the average number of scattering events *N*, using model based *Α*(*N*) = α/α_*1*_, and 1/*τ*_*c,1*_ = (1/*τ*_*c*_)/*Α*(*N*). In both (**a,b**) one data point was excluded as an outlier (not shown). Vertical error bars represent 95% CI of the multi-exposure curve fit and horizontal error bars represent the standard deviation in reference flow velocity measurements from conventional SDF images. The slope, or *α*_*1*_, is 0.20 ± 0.07 (95% CI) and 0.39 ± 0.15 for chick embryo respectively human RBCs, and the theoretical prediction for *α*_*1*_ is 0.27 respectively 0.38.

We assessed *in vivo* decorrelation times by imaging the readily available microcirculation of the chorioallantoic membrane of a chick embryo grown *ex ovo*. Multi-exposure SDF-LSCI and conventional SDF frames were recorded for 35 unique vessels for which RBC tracking was possible, to independently estimate *τ*_*c*_ (by LSCI) and *V* (by SDF) of the same vessels. Compared to controlled phantom experiments, first it is essential to account for ‘offset’ decorrelation due to dynamic scattering of photons outside the focal plane or caused by muscle movements^21^ (see Methods). Second, the average number of scattering events varies per vessel diameter, estimated from Monte Carlo simulations (Supplementary Section IV). Therefore, for each vessel a unique *α*(*N*, *μ*_*s*_, *p*(***q***)) can be found. We assume that for the microcirculation, scattering coefficient *μ*_*s*_ and phase function *p*(***q***) are invariant and we can write *α*(*N*). This allows rescaling of each *τ*_*c*_ to *N* = 1 by a model-based *in vivo* scaling factor *Α*(*N*) = *α*/*α*_*1*_, denoting the parameters rescaled for single scattering *α*_*1*_ and *τ*_*c,1*._ The scaling factor *A*(*N*) calculated for chick embryo blood vessels is shown in Supplementary Fig. 5. In Fig. 6a 1/*τ*_*c*_, (top panel) and the rescaled 1/*τ*_*c,1*_ (bottom panel) are plotted versus blood flow velocity for the chick embryo blood vessels. The correlation coefficient of the linear fit of 1/*τ*_*c*_ and 1/*τ*_*c,1*_ vs. *V* improved after rescaling from r = 0.11 to r = 0.74, respectively. The slope of the rescaled linear fit gave *α*_*1*_ = 0.20 ± 0.07 (95% CI), close to theoretical *α*_*1*_ = 0.27 with an uncertainty range of [0.24–0.30] calculated by varying the size ±5% and refractive index *n*_*RBC*_ ±1% (Supplementary Table 1). Next, we reassessed the human sublingual microcirculation measurements acquired previously (14 unique vessels with known flow velocities)^21^ by model-based rescaling of *τ*_*c*_ using *A*(*N*) from Fig. 2 for Hct = 30%. The correlation coefficient increased from r = 0.48 to r = 0.87 between 1/*τ*_*c*_ (Fig. 6b, top panel) and the rescaled 1/*τ*_*c,1*_ data (Fig. 6b, bottom panel), respectively, vs. flow velocity. The *in vivo α*(human) was 0.41 ± 0.50 (95% CI) for the original data and *α*_*1*_ was 0.39 ± 0.15 (95% CI) for the rescaled data, near identical to the theoretical prediction for α_*1*_ of 0.38 with an uncertainty range of [0.34–0.41] calculated by varying the size ±5% and *n*_*RBC*_ ±1%. In both (a) and (b) in Fig. 6 one data point was excluded as an outlier.

**Figure 7:**
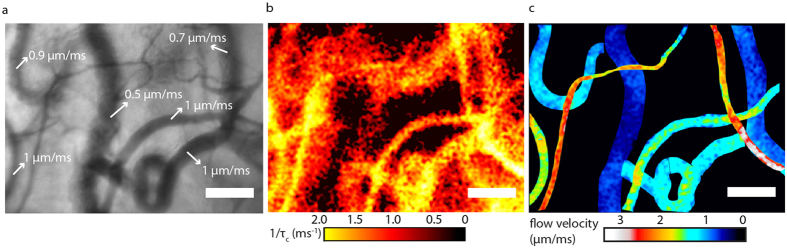
Human microcirculatory flow velocity mapping. **(a)** Conventional SDF image where the contrast is based on absorption differences between RBCs and tissue. Flows below 2 mm/s can be measured by RBC tracking. **(b)** 1/*τ*_*c*_ map of the same microcirculation region obtained with multi-exposure SDF-LSCI after correction for *τ*_*c,offset*_, contrast is obtained by perfusion dynamics. **(c)** Map of LSCI-derived blood flow velocities after correction for *τ*_*c,offset*_ and *A*(*N*), and masking of selected blood vessel contours. The scale bar is 100 μm.

To illustrate the quantitative imaging ability of laser speckle flowmetry we constructed a 1/*τ*_*c*_ map (representing the basic LSCI outcome) and a blood flow velocity map as shown in Fig. 7 and Supplementary Movie 1 (human) and Supplementary Movie 2 (chick embryo), following the practical steps outlined in Supplementary Fig. 2 and Supplementary Section VII.

## Discussion

In this Article we demonstrate non-invasive quantitative blood flow velocity measurements *in vivo*, using a relatively simple and available technique based on LSCI. LSCI suffers from a debated quantitative relationship with blood flow, velocity or tissue perfusion^22^,^23^ and a lack of calibration of the inverse relationship between *τ*_*c*_and flow velocity quantified by the proportionality constant *α*^25^. Several predictions for *α* have been suggested in literature, but disagree in their absolute value and physical dependence^26^,^27^,^28^,^46^. The results of our study confirm that scatterer size and volume fraction influence the relationship between 1/*τ*_*c*_ and *V*^28^. To quantitatively understand this relation we employed a theoretical model based on optical and physical properties of the scatterers, where *α* is particularly influenced by the scattering phase function and multiple scattering effects. Our assumption that the scattering properties of RBCs in the microcirculation are invariant allows for rescaling by the number of scattering events, enabling quantitative *in vivo* blood flow velocity measurements. This has previously been implemented empirically by rescaling *τ*_*c*_ by a weighting term that is proportional to the vessel diameter^16^, or local absorption properties related to blood content^47^, though in a qualitative fashion. The quantity 1/*τ*_*c*_ is essentially related to flow velocity and vessel diameter/blood content, therefore a strong linear correlation with *V* alone is not expected, however, by correcting for volume fraction of RBCs the linear correlation is much improved as also evidenced by the reduction in 95% CI range for α_*1*_. We estimated the average number of scattering events with Monte Carlo simulations and related their distribution to the Mie-Percus-Yevick (MPY) scattering model of whole blood. This approach resulted in an excellent agreement between theory and experiment, validating *α* and its correction factor for multiple dynamic scattering, *A*(*N*), *in vivo* for microcirculatory blood flow velocities and confirming that quantitative measurement of flow velocity by LSCI is feasible. Conveniently, vessel diameters and vessel density can be estimated from the images, allowing quantitative mapping of both blood flow and velocity and estimating tissue perfusion using laser speckle flowmetry.

In Figs 4 and 5 we systematically varied the size and volume fraction of flowing polystyrene spheres and compared the experimental results to theoretical predictions. In Fig. 4b α for 5, 7 and 10 μm particles (2.5 vol%) is in good agreement with our predictions, but for smaller scatterers (0.6, 1 and 2 μm) *α* is overestimated by theory. For the small scatterers, which are below the resolution limit of the system, the measured *α* seems to saturate to a value of 2–2.5 μm^−1^ in our system. A similar value for *α* was found in our previous report, using 2.5 vol% Intralipid® as flowing fat emulsion with sub-resolution effective scatterer size^21^. Supplementary Fig. 7 shows that saturation of *α* is expected for small scatterers, albeit at much higher values. Moreover, *α* does not show dependence on volume fraction for these small spheres (Fig. 5), suggesting that *α* in this case is predominantly determined by system properties rather than sample properties^24^,^26^. On the other hand, from Supplementary Table 1 and Fig. 5b, all experiments with small particles involve a high *μ*_*s*_ and thus a large number of dynamic scattering events so that an alternative explanation is that our theory overestimates decorrelation by multiple scattering when the number of events get large. A satisfactory theoretical explanation for either possibility has hitherto not been found.

In our *in vivo* SDF-LSCI geometry, RBC size exceeds the resolution limit. Although in whole blood (high *μ*_*s*_) multiple scattering is present for most vessels (our data set: vessel diameter = [10–55 μm]) the average number of scattering events *N* = [1] was low, making saturation of *α* unlikely. Numerically simulating *N in vivo* and applying the theoretical model to the optical properties of blood allowed to rescale the obtained *τ*_*c*_ for multiple scattering resulting in a high linear correlation between 1/*τ*_*c,1*_ and flow velocity. In addition, the measured *α*_*1*_ is in perfect agreement with the theoretical prediction. Though *in vivo α* could only be validated against low flow velocities (<2 mm/s) measured by conventional SDF flowmetry, the *in vitro* experiments verified *α* up to 15 mm/s indicating that LSCI is capable of measuring high microcirculatory flow velocities as well. Extending the multi-exposure regime to shorter exposure times will increase the maximum flow velocity limit further.

Our multiple scattering model in whole blood based on the MPY approach matches the experimental results well, therefore the *in vivo* scaling factor *Α*(*N*) in Fig. 2, can be used as a practical guideline to quantitative laser speckle flowmetry when *N* can be estimated. To recapitulate, dynamic scattering by multiple moving particles yields a different time-constant *τ*_*c*_ compared to single scattering. *Α*(*N*) is simply the ratio of these time constants (*A*(*N*) = *τ*_*c,1*_/*τ*_*c*_ or *A*(*N*) = α/α_1_) at a certain value for *N*, relating *V* to *τ*_*c*_*in vivo* via 1/*τ*_*c*_ = α_1_*Α*(*N*) ×*V*. Thus, the practical realization of quantitative laser speckle flowmetry necessitates the estimation of *N* from the geometry of blood vessels. Then, *τ*_*c,1*_ is calculated according to 1/*τ*_*c,1*_ = (1/*τ*_*c*_)/*Α*(*N*) and the flow velocity estimated from 1/*τ*_*c,1*_*≈* [0.38 ± 0.04]*V*. For comparison to conventional LSCI approaches we also plotted *Α*(*N*) for Gaussian and Lorentzian shape of *g*_*1*,_(*τ*) (Fig. 2, dashed lines), calculated using a normal distribution for *p*_*N*_(*n*). Rescaling for multiple scattering is thus highly model dependent, where the Lorentzian (*A*(*N*) ~ *N*) and the Gaussian (*A*(*N*) ~ *√N*) model result in an underestimation or overestimation of *V*, respectively.

We use the analytical model of Supplementary equation (12) to fit experimental *K*(*T*) vs. *T* curves, based on the assumption that this model accurately retrieves the actual decorrelation time *τ*_*c*_, even though our derived *g*_*1*_(*τ*) is not necessarily purely Gaussian. We verified the validity of this approach by calculation (Supplementary Section XI). It is shown that the current model and the Gaussian model approximate each other sufficiently, especially for *N* > 1, by examining the final *K*(*T*) curve. We therefore conclude that assuming a Gaussian ACF, thereby making the fit model for *K*(*T*) analytical and practical, results in an acceptably small error in *τ*_*c*_ estimation. This result does not imply that scaling factors *A*(*N*) should be based on Gaussian approximations to *g*_*1*_(*τ*) as evidenced by Fig. 2. We therefore scale our *in vivo* measured *τ*_*c*_ to the value *τ*_*c,1*_ (for single scattering) using the MPY model-based factor *A*(*N*). This highlights an important finding of our study: although the *in vivo* estimation of *τ*_*c*_ is valid using a Gaussian form for *g*_*1*_ and its often-used expression for *K*(*T*)^21^,^24^,^42^, the subsequent rescaling of *τ*_*c*_ to *τ*_*c,1*_ is recommended using our MPY-based *A*(*N*) in Fig. 2, to prevent over- or underestimation of *V* when using the Gaussian or Lorentzian model respectively. A recent study by Kazmi *et al.*^48^ applied the *N*-scattering correction using the more straightforward Gaussian approach and found an improved correlation coefficient between 1/*τ*_*c*_ and *V*, as compared to uncorrected values. However, the absolute at certain still differed by a factor of 2 for different animals. This good *relative* relationship between both quantities can also be explained by our modelling in Fig. 2: comparing our model to the Gaussian based *A*(*N*) results in similar *relative* correction factors between *V* and *τ*_*c*_. Thus, in order to absolutely quantify flow velocity (and thus other important physiological parameters like perfusion), we believe that our MPY-based rescaling factor, and its related *p*_*N*_(*n*), are crucial. We emphasize that *τ*_*c*_ *in vivo* is subject to additive decorrelation sources along the photon path through tissue^21^, referred to as the ‘offset’ correction, which should be estimated and corrected accordingly.

Introducing the offset-decorrelation correction to conventional LSCI analysis yields a quantitative decorrelation map (Fig. 7b) and subsequently applying the model-based scaling factor and α_1_ yields a quantitative blood velocity map (Fig. 7c). We note that more advanced image analysis algorithms (discussed in Supplementary Section VII) will reduce the small differences between flow velocities found using RBC-tracking (Fig. 7a) and quantitative LSCI (Fig. 7c). The offset correction was introduced in our previous *in vivo* SDF-LSCI validation study^21^. The offset correction holds if all decorrelation sources are statistically independent. Only in that case is the total decorrelation function given by the product of the decorrelation functions of each process^21^,^24^. Moreover, our correction assumes that all decorrelation processes are well described with Gaussian ACFs and that pixels representing tissue sample only the offset decorrelation, while the pixels representing vessels sample the offset *and* additional flow decorrelation. The first assumption is assessed in the previous paragraph. The last assumption is substantiated by the low scattering of the sublingual mucosal non-keratinized top layer resulting in a high probability that photons are derived from the focal plane (the high quality SDF-images of flowing RBCs substantiate this). However, in highly scattering tissues such as skin the offset correction warrants further study. To illustrate the importance of the offset-correction in the sublingual microcirculation, we plotted the data from Fig. 6 without the offset correction in Supplementary Fig. 8, i.e. 1/*τ*_*c,total*_ and 1/*τ*_*c1,total*_ vs. *V*. As chick embryo vessels are embedded in a weakly scattering medium (egg-white) and have low vessel density, the offset decorrelation times are long compared to flow decorrelation times and therefore have less influence. For human vessels on the other hand, not applying the offset correction reduces the linear correlation coefficient between 1/*τ*_*c,1,total*_ and *V* substantially and the resulting values for *α*_*1*_ deviate from theoretical values (see Supplementary Fig. 8). Thus, in human microcirculation beds, decorrelation events from vessels outside the focal area have a substantial influence on the detected *τ*_*c*_’s in laser speckle contrast images^49^ and the offset-correction is necessary.

The uncertainty in the estimation of *N* with Monte Carlo simulations is due to uncertainties in the included optical properties and fidelity of the simulated measurement geometry. Our phantom experiment offers an alternative way to validate *N* as described in Supplementary Section X and Supplementary Fig. 9. The latter figure shows the dependence of parameter *ρ* on the scattering coefficient of the dynamic medium. Naturally, the measurement geometry also influences *ρ* (e.g. due to different static/dynamic medium properties, tube diameter, etc.) thus *ρ* needs appropriate estimation/calibration. *In vivo*, *ρ* is influenced by imaging geometry, vessel diameter, vessel depth and Hct values and can be estimated from measurements at long exposure times. When neglecting to appropriately estimate *ρ* the resulting value for *τ*_*c*_ can deviate majorly^16^,^31^. Estimation of *N in vivo* using Monte Carlo simulations depends on the geometrical and optical properties such as vessel depth, diameter and *μ*_*s,blood*_. Varying the vessel depth between 0.1 and 0.4 mm and the Hct between 30% and 45% resulted in comparable values for *N* (Supplementary Fig. 4), indicating that our estimation of *N* is robust. In addition, calculating *α*_*1*_ using this variation in *N* values influenced *α*_*1*_ by less than ±0.02 for microcirculatory vessel diameters. Alternatively, low-coherence interferometric set-ups allow path-length resolved speckle signals to be acquired^50^,^51^,^52^, providing better estimation of *N* albeit without the simplicity of the LSCI approach.

To make our results more widely applicable we performed Monte Carlo simulations for a conventional speckle imaging set-up where wide field laser light illuminates tissue at an angle and the backscattered light is captured by a camera directly above the tissue^10^,^13^,^14^,^15^,^16^,^53^. In this geometry very similar *N* values for dynamic detected photons were found for both *in vitro* and *in vivo* optical properties (Supplementary Fig. 4), which generalizes our multiple scattering scaling. Practically, combining the results from Monte Carlo simulations for *N*, calculation of *A*(*N*) and the theoretically and experimentally derived value for *α*_*1*_ the relationship between 1/*τ*_*c*_ and *V* simplifies to 1/*τ*_*c*_ = 0.6*V*, 1/*τ*_*c*_ = 1.2*V* and 1/*τ*_*c*_ = 2.0*V* for 20, 50 and 100 μm microcirculatory vessels, respectively.

The approach to calculate *g*_*1*_ for multiple scattering has been recognized in literature^50^,^52^,^54^, explicitly in the LDF^28^ and DWS^34^ field. The differences in the treatment of multiple scattering and modelling *g*_*1*_ between LDF, DWS and our approach are discussed in Supplementary Section XII. Key challenges are to find a valid model for *g*_*1*_ and a valid expression for *p*_*N*_(*n*) in the specific geometry. Here we have shown that our experimental *α*_*1*_ = 0.39 (and estimation of *p*_*N*_(*n*)) matches very well with theory (*α* = 0.38) and falls in between predictions from the LDF framework (*α*_*1,LDF*_ = 0.36) and DWS framework (*α*_*1,DWS*_ = 0.54).

The experimental and theoretical findings in this study improve the quantitative flowmetry capabilities of LSCI and show that the clinically relevant parameters blood flow, velocity and tissue perfusion, can be quantitatively represented in SDF-LSCI microcirculation images, escaping the doctrine of qualitative and relative flow measures (Supplementary section XIII). This enables SDF-LSCI to quantitatively study microcirculation within and between organs and organisms and during the course of disease and therapy. In essence, for quantitative *in vivo* speckle flowmetry reliable estimation of *τ*_*c*_, correction for ‘offset’ decorrelation^21^, and estimation of vessel diameter to correct for multiple scattering are key ingredients. We recommend to consider the influence of system resolution on *α*, for example when choosing flowing scattering solutions in phantom experiments. The highly vascularised chorioallantoic membrane of the chick embryo can be a practical calibration model as an intermediate between *in vitro* and *in vivo* validation.

## Methods

### Data acquisition

A clinical microcirculation imager based on SDF microscopy (Microscan, Microvision Medical, The Netherlands) was modified to provide illumination with laser light (HeNe, 632.8 nm). Four multimode fibres surround a central imaging pathway (5× magnification, 4.2 μm resolution ) (Supplementary Fig. 1)^21^. Back-reflected light forms a speckle pattern on an 8-bit monochrome camera (IEEE 1394, Guppy F-080B, Allied Vision Technologies, Germany) with a field of view of 1 × 0.7 mm on 1024 × 768 pixels. The minimal speckle diameter was 2.2 pixels ≈10.2 μm^55^. To obtain a multi-exposure curve, we applied an exposure time range of 0.3–30 ms (*in vitro*)/100 ms (*in vivo*) using neutral density filters to prevent overexposure (Supplementary Fig. 1). For conventional SDF imaging broad band green light (530±20 nm) was coupled into the fibres to provide absorption-based contrast between flowing RBCs and surrounding tissue, enabling RBC flow measurement^56^. During *in vivo* data acquisition the SDF and SDF-LSCI modes were alternated to measure the flow velocity and decorrelation time of the same region^21^.

### *In vitro* flow phantom

We designed a flow phantom consisting of a polymer tube (diameter 0.2 ± 0.03 mm; depth 0.3 mm ± 0.03 mm) embedded in silicone elastomer (Sylgard® 184 Silicone Elastomer DOW/Corning, US) mixed with titanium dioxide (TiO_2_, anatase form, Sigma Aldrich, US) in the concentration [TiO_2_] = 1 mg/ml to mimic tissue scattering^57^. The tube was connected to a 0.5 ml syringe, slowly pressed by a syringe pump (Harvard model PHD2000, US) providing a flow range of 0.1–20 mm/s. The flowing media consisted of polystyrene spheres in water with diameters 0.6, 1, 2, 5, 7 and 10 μm (Kisker-Biotech, Germany, stock volume fraction 2.5 vol%). For 1, 2 and 5 μm particles we prepared different volume fractions in the range 0.6–7 vol%. The range of scattering coefficients *μ*_*s*_ was 7–150 mm^−1^ as specified in Supplementary Table 2. Flow series were repeated 2 or 3 times per sample. Low flow (<1 mm/s) measurements on large spheres (>2 μm) were excluded due to precipitation of scatterers.

### *In vivo* microcirculation

We recorded *in vivo* SDF-LSCI image frames from the microcirculation of a chick embryo at embryonic day 9, grown *ex ovo*^58^. The imaging tip of the integrated SDF-LSCI device was gently put in contact with the chorioallantoic membrane tissue to prevent disruption of blood flow. The human sublingual microcirculation SDF-LSCI dataset was previously obtained^21^ and analyzed using the current MPY-based model. For the sublingual microcirculation the device was hand-held, while it was secured in a stand for chick embryo microcirculation imaging.

### Data analysis

*In vitro*: Speckle contrast *K* was calculated from the raw speckle images according to Supplementary section IIId, equation (10) over a local region of 10 × 10 pixels. For each exposure time *T*, minimally 200 *K*-values were selected in the center of the tube (standard deviation in *K* < 5%). Next, nonlinear curve-fitting of Supplementary equation (12) to the multi-exposure curves was performed to find *τ*_*c*_. Here, *β*_*M*_ and *ρ* were a-priori estimated at T << *τ*_*c*_ (static phantom) and at T >> *τ*_*c*_ (flow >15 mm/s and T > 10 ms) respectively, leaving *τ*_*c*_ the only fit parameter in Supplementary equation (12). *β*_*M*_ is expected to be constant throughout and was found to be 0.40 ± 0.02. Specifically, *ρ* was estimated for each scatterer size and volume fraction and kept constant for different flow velocities. The goodness of nonlinear fit <R_adj_^2^> averaged over all fits of *K*(*T*) vs. *T* was 0.98 ± 0.02.

*In vivo:* To calculate *K* a spatiotemporal local region of 7 × 7 × 20 pixels was applied to optimize the *in vivo* spatiotemporal resolution and minimize the standard deviation in in *K* (<7%)^21^. Minimally 25 *K* values were obtained per vessel (or adjacent tissue region) and *T*. The exposure time range was extended to longer T [0.5–100 ms] and *ρ* and *β*_*M*_ were a-priori estimated at long and short *T* respectively. In the subsequent curve fit both were constraint within 5% of their estimated value, and *τ*_*c*_ was unconstraint. The goodness of nonlinear fit <R_adj_^2^> averaged over all fits was 0.99 ± 0.01. The *in vivo* decorrelation times (*τ*_*c,total*_) were corrected for statistically independent sources of decorrelation via *τ*_*c,offset*_ measured from adjacent tissue (similar to *τ*_*c,total*_) as described in Supplementary equation (13)^21^. Reference flow measurements (maximal measurable flow 2 mm/s) were obtained from SDF-mode images using commercially available software (AVA3.0, Microvision Medical, The Netherlands)^56^. *In vivo* flow measurements were repeated minimally 3 times per vessel and vessels with a large variation in flow were excluded. To correct the decorrelation times for multiple scattering the number of scattering events *N* in the vessel were estimated using Monte Carlo simulations as described in Supplementary Section IV yielding the *in vivo* scaling factor *Α*(*N*) = α(*N*)/α_*1*_, where *α*(*N*) was theoretically derived using the optical properties of blood (Supplementary Table 1) for *N* scattering events, and *α*_*1*_for single scattering. Finally, 1/*τ*_*c*_ was rescaled according to 1/*τ*_*c,1*_ = (1/*τ*_*c*_)/*Α*(*N*).

### Ethics Statement

The chick embryo experiments are exempt from animal ethics committee approval, in accordance with the EU directive/Dutch law on animal experiments (WOD). For this Article we have not done additional human experiments as we have re-analyzed previously obtained data from human microcirculation (ref. 21).

## Additional Information

**How to cite this article**: Nadort, A. *et al.* Quantitative blood flow velocity imaging using laser speckle flowmetry. *Sci. Rep.* **6**, 25258; doi: 10.1038/srep25258 (2016).

## Supplementary Material

Supplementary Movie 1

Supplementary Movie 2

Supplementary Information
