# Supplementary material for: Quantitative blood flow velocity imaging using laser speckle flowmetry
Source: Sci Rep. 2016 Apr 29;6:25258. doi: 10.1038/srep25258 (PMC4850477; doi:10.1038/srep25258)
Supplement: Supplementary Information [file srep25258-s3.pdf]

Quantitative blood flow velocity imaging using laser speckle flowmetry

Annemarie Nadort,[1,2] Koen Kalkman,[1] Ton G. van Leeuwen,[1] and Dirk J. Faber[1*]

[1] Department of Biomedical Engineering and Physics, Academic Medical Center, University of Amsterdam, P.O. Box 22700, 1100 DE Amsterdam, The Netherlands

[2] ARC Centre of Excellence for Nanoscale BioPhotonics, Macquarie University, Sydney 2109, NSW Australia

**d.j.faber@amc.uva.nl*

Supplementary Information

Supplementary Information

I - Sidestream Dark Field and Laser Speckle Contrast Imaging (SDF-LSCI) set up

To enable absolute blood flow measurements and estimation of τ_c of the same *in vivo* vessels we modified a clinical microcirculation imager (Microscan, Microvision Medical, The Netherlands) to allow illumination by different light sources[1]. The conventional system provides illumination by six green LEDs at the tip of the lens tube, which were replaced by four optical fibres (POF ESKA, fibre core 980 µm, NA 0.5). The fibres are directed through the imager alongside the lens tube, which is covered with a sterile cap and can be brought into contact with the tissue of interest. In SDF-LSCI mode, as is shown in Supplementary Fig. 1, laser light (632.8 nm, He/Ne, Spectra Physics, US) is split into four beams by three 50:50 beam splitters and coupled into the four multimode fibres. Raw speckle frames with different exposure times are captured by a monochrome camera at 30 frames per second (IEEE 1394, Guppy F-080B, Allied Vision Technologies, Germany), controlled by self-written software (LabVIEW, National Instruments, US). For exposure times above 33 ms obviously the frame rate is less. Overexposure is prevented by a neutral density filter wheel in front of the laser. In conventional SDF mode the fibres are illuminated by four LEDs (530 ± 20 nm). Conventional SDF images show good contrast between tissue and highly absorbing red blood cells (RBCs). Conventional and SDF-LSCI mode were consecutively employed to image the same microcirculation area. The focal plane can be translated between tissue surface and 0.4 mm depth and the optical magnification is 5x.

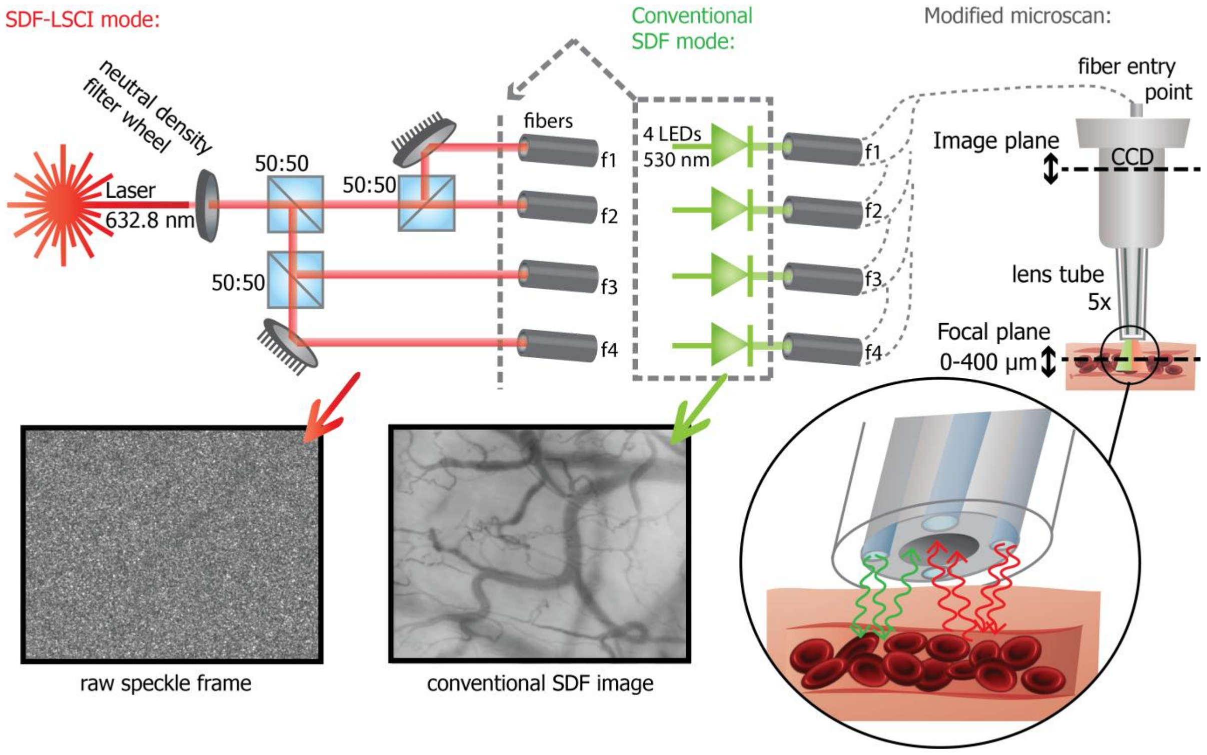

Supplementary Fig. 1 | Dual-mode non-invasive microcirculation imaging set-up. SDF-LSCI mode: speckle contrast due to reflection of coherent laser light by flowing RBCs and 'static' tissue. Conventional SDF mode: contrast due to absorption of green light by flowing RBCs. See text above for more details.

II - Flow chart for quantitative laser speckle flowmetry

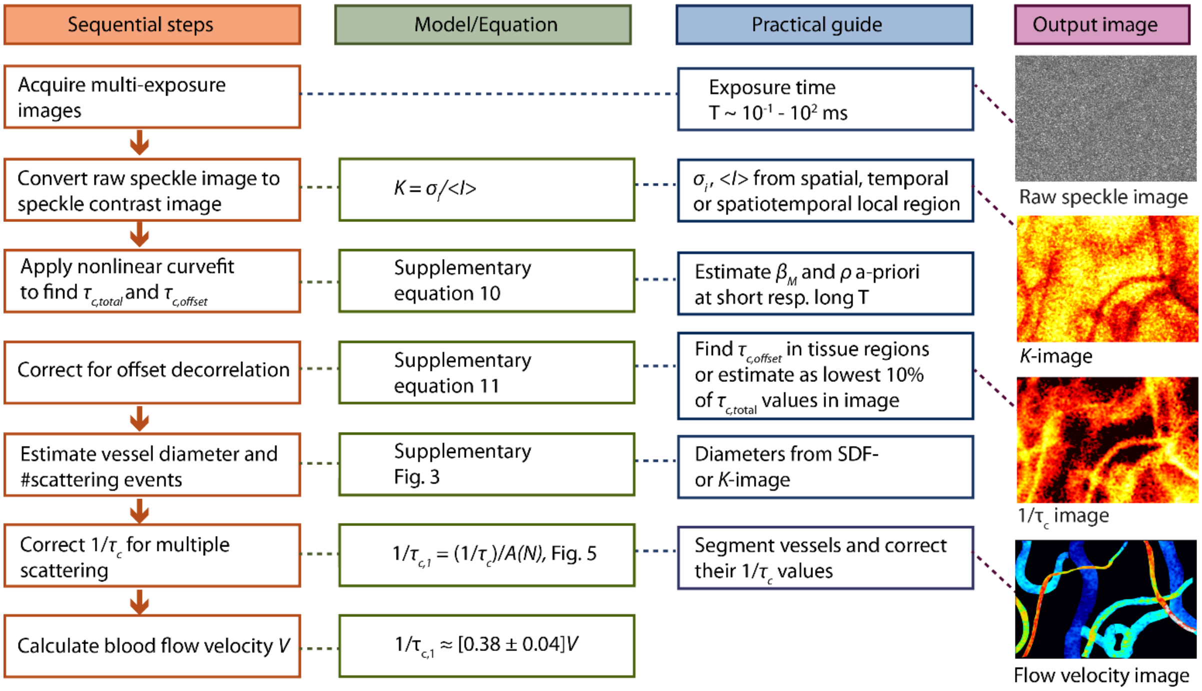

Supplementary Fig. 2 | Flow chart for quantitative laser speckle flowmetry. This work discusses the sequential steps, theoretical model and practical procedure to quantifying blood flow velocities *in vivo* using laser speckle contrast imaging (LSCI). This flow chart serves as a guideline for the reader.

III – Theory

IIIa - Optical and physical properties of the dynamic medium

We briefly review the optical properties relevant to our application. We model our phantom and tissue as a discrete random medium of hard spheres (i.e. occupying non-overlapping volumes). The differential scattering cross section $\sigma_{sca}(\boldsymbol{q})$ describing the angular distribution of scattered light by a single sphere, is calculated using Mie (M) theory[2]. Here $\boldsymbol{q}=\boldsymbol{k}_{scat}-\boldsymbol{k}_{in}$ is the scattering vector with magnitude $|\boldsymbol{q}|=2k\sin(\theta/2)$; k is the wavenumber $2\pi/\lambda$ and θ is the scattering angle. To account for inter-particle correlation effects ("dependent scattering"), we calculate the structure factor $S(\boldsymbol{q})$ using the Percus-Yevick (PY) equation[3] for hard spheres[4]. The scattering coefficient μ_s, cross section σ_{sca} and scattering phase function $p(\boldsymbol{q})$ are then found, respectively, as:

$$\mu_{s,MPY} = \eta\sigma_{sca,MPY} = \eta 2\pi\int_0^{2k} \sigma_{sca,MIE}(\boldsymbol{q}) S_{PY}(\boldsymbol{q}, f_v)\boldsymbol{q}d\boldsymbol{q} \tag{1a}$$

$$p_{MPY}(\boldsymbol{q}) = \frac{\sigma_{sca,MIE}(\boldsymbol{q}) S_{PY}(\boldsymbol{q}, f_v)}{\sigma_{sca,MPY}} \tag{1b}$$

Here $\eta = f_v/V_p$ is the particle number density in the medium; with f_v the volume fractions of particles with volume V_p. The structure factor is given by the Fourier transform of the *pair correlation function* PCF(r) which is interpreted as the distribution of distances r between particle pairs. For dilute solutions of hard spheres of diameter D, PCF(r) = 0 when $r < D$ and unity otherwise. For higher volume fractions PCF(r) shows damped oscillatory behaviour, with increased probability of finding pair separations at multiples of D and decreased probability in between. In the limit $r\rightarrow\infty$; PCF(r) goes to unity. The structure factor $S(\boldsymbol{q})$ is approximately constant at unity for low volume fractions. Non-unity $S(\boldsymbol{q})$ at higher values of f_v cause an angular redistribution of scattered light (Supplementary equation (1b)) as well as non-linear scaling of the scattering coefficient. Analytical forms of $\sigma_{sca,MIE}(\mathbf{q})$ and $S_{PY}(\mathbf{q})$ are not available but may be computed by algorithms published in ref. 2 for Mie theory and ref. 5for Percus-Yevick, respectively.

IIIb - Dependence of τ_C on the optical properties of the dynamic medium

Consider an ensemble of scatterers (indexed j) each moving with velocity $\boldsymbol{V}_j$ in a vessel within an otherwise static, scattering medium. The velocity distribution is given by $p(\boldsymbol{V})$. The normalized electric field autocorrelation (ACF) function $g_1(\tau) = \langle E(t+\tau)E^*(t)\rangle/\langle|E(t)E^*(t)|^2\rangle$, for a single scattering event from moving particles is[6]:

$$g_{1,\text{single}}(\boldsymbol{q},\tau) = \left\langle \sum_j \exp\left(-i\boldsymbol{q}\cdot\boldsymbol{V}_j\tau\right)\right\rangle \tag{2}$$

When light undergoes many scattering events before reaching the vessel, the propagation direction becomes isotropic. Consequently, the direction of the scattering vector $\boldsymbol{q}$ with respect to the velocity vector $\boldsymbol{V}$ also becomes isotropic, and the velocity distribution depends only on the magnitude $|\boldsymbol{V}|=V$. We assume a Gaussian-shaped speed distribution $p(V) = \sqrt{(2/\pi)}\,(3/V_0)^{3/2}\exp(-3V^2/2V_0^2)$; where V_0 is the average flow velocity. Averaging over $p(V)$ yields[6-8]:

$$g_{1,\text{single}}(\boldsymbol{q},\tau) = \exp\left(-\boldsymbol{q}^2V_0^2\tau^2/6\right) \tag{3}$$

In the following, we drop the subscript '0' on velocity. The average over $\boldsymbol{q}$ is calculated as:

$$g_{1,\text{single}}(\tau) = \int_0^{2k} p_{MPY}(\boldsymbol{q})\exp\left(-\boldsymbol{q}^2V^2\tau^2/6\right)\boldsymbol{q}d\boldsymbol{q} \tag{4}$$

The angular weighting by the phase function of Supplementary Equation (1b), introduces dependence on the optical and physical properties in g_1 (and thus in τ_C).

IIIc Multiple scattering

Supplementary equation (4) describes the electric field decorrelation due to single scattering events. If subsequent scattering events are assumed to be statistically independent, the ACF becomes $[g_1(\tau)]^n$ where n is the number of scattering events from moving scatterers. The total correlation function follows after weighting with the appropriate distribution of n:

$$g_1(\tau) = \sum_{n=1}^{\infty} p_N(n)\left[g_{1,\text{single}}(\tau)\right]^n \tag{5}$$

Because the scattering events take place at the particles, the distribution $p_N(n)$ in the measurement volume follows the microscopic distribution of the number of particles $p_N(\eta_{\text{micr}})$ in the dynamic medium. If the particles positions are independent of each other, $p_N(\eta_{\text{micr}})$ and $p_N(n)$ are given by the Poisson distribution[9], with equal mean and variance of $\langle\eta_{\text{micr}}\rangle = \sigma^2_{\eta\text{micr}} = \eta$ (the global average density); and of $\langle n\rangle = \sigma^2_n = N$, respectively. When correlations between the particle positions are present, e.g. when PCF(r)≠1, the Poissonian distribution model is no longer valid. Although the exact distribution is unknown, the relation between mean (N) and variance (σ_n^2) is determined by the PCF as[10,11]:

$$\frac{\sigma_n^2}{N} = 1 + \eta\int\left[PCF_{PY}(r) - 1\right]dr = \frac{\left(1 - f_V\right)^4}{\left(1 + 2f_V\right)^2} \tag{6}$$

Where the right-hand side is valid for the PY equation of PCF(r) for hard spheres. In our present analysis, we assume $p_N(n)$ follows a normal distribution where the relation between mean and variance is given by Supplemenatary equation (6). Here,

$$p_N(n) = \frac{1}{\sqrt{2\pi\sigma_N^2}} e^{-\left((n-N)^2/2\sigma_N^2\right)} \tag{7}$$

Parameter N is estimated from Monte Carlo simulation of our experimental geometry (Supplementary Section IV) The ACF $g_1(\tau)$ is an exponentially decaying function of time, parameterized by the characteristic timescale τ_c (decorrelation time) which is evaluated from [6,9]:

$$\tau_c = \int_0^{\infty} g_1(\tau)d\tau \tag{8}$$

Following the above outlined exercise, g_1 can be plotted according to the set of optical and physical properties (including N) and the proportionality constant α can be derived as $1/\tau_c$ at $V = 1$. The ACF calculated using the above formulae is of Gaussian form (because all involved functionals, i.e. phase function, velocity distribution and distribution of the number of scattering events, are of (near) Gaussian shape). In the following, we therefore assume that $g_1(\tau) = \exp(-0.25\pi(\tau/\tau_c)^2)$ for mathematical convenience of the procedure to extract τ_c from spatial speckle contrast measurements (Supplementary Section IIId). The validity of this approximation is discussed in Supplementary Section XI.

Our aim is to quantify the relationship $1/\tau_c = \alpha V$ *in vivo*. For microcirculatory blood flow, the measured τ_c can be due to scattering from a single flowing RBC (supplementary equations (4) and (7)) or from multiple flowing RBCs (supplementary equations (5) and (7)), referred to as $\tau_{c,1}$ and τ_c, for single and multiple scattered light, respectively.

Thus, α relating V to τ_c also needs to reflect single, α_1, or multiple, α, scattering. As a practical approach we introduce the rescaling factor $A(N)$, defined as:

$$A(N)=\frac{\alpha}{\alpha_1}=\frac{\tau_{c,1}}{\tau_c}=\frac{\int_0^\infty g_{1,\text{single}}(\tau)d\tau}{\int_0^\infty \sum_{n=1}^{\infty} p_N(n) g_{1,\text{single}}^n(\tau)d\tau} \tag{9}$$

The functional dependence of $A(N)$ on N for human blood is plotted in Fig. 2 (Article) and Supplementary Fig. S5 for chick embryo blood. The general formula relating V to τ_c then becomes $1/\tau_c = \alpha_1 A(N) \times V$.

IIId - Spatial assessment of temporal speckle dynamics

Laser Speckle Contrast Imaging (LSCI) assesses sample dynamics from spatial measurement of speckle contrast, defined as the ratio of the standard deviation (σ_i) to the mean ($<I>$) of the intensity:

$$K=\frac{\sigma_i}{\langle I\rangle} \tag{10}$$

This quantity is related to sample dynamics through temporal integration of the ACF $g_1(\tau)$ as obtained from Supplementary equation (5)[9]:

$$K(T)=\beta_M^{1/2}\left[\frac{2}{T}\int_0^T\left(1-\frac{\tau}{T}\right)\left|g_1(\tau)\right|^2 d\tau\right]^{1/2} \tag{11}$$

Here, T is the exposure time of the camera and β_M is a measurement-geometry specific constant that can be calibrated[1]. In the general case of flow in a single vessel or channel, $g_1(\tau)$ is assumed to have a Gaussian form $g_1(\tau) = \exp(-0.25\pi(\tau/\tau_c)^2)$ as described in Supplementary Section Ic. For Brownian motion, $g_1(\tau)$ takes an exponential form (corresponding to a Lorentzian lineshape): $g_1(\tau) = \exp(-(\tau/\tau_c))$. A sampled biological tissue volume likely consists of both static and dynamic scatterers. The influence of the static component on $g_1(\tau)$ can be taken into account[12-14] by introducing a dependence on $\rho = I_f/(I_f + I_s)$, with I_f the detected intensity of the fluctuating scattered light and I_s the detected intensity of the light scattered by static components. The speckle contrast Supplementary equation (11) can be solved analytically for the Gaussian form of $g_1(\tau)$ to give[12,13]:

$$K(x)=\beta^{1/2}\left[\rho^2\frac{\exp\left(-2(x)^2\right)-1+\sqrt{2\pi}x\,erf(\sqrt{2}x)}{2(x)^2} +2\rho(1-\rho)\frac{\exp\left(-(x)^2\right)-1+\sqrt{\pi}x\,erf(x)}{(x)^2}+(1-\rho)^2\right]^{1/2}+C_{noise} \tag{12}$$

where $x = \sqrt{\pi}T/2\tau_{c,total}$ and C_{noise} an added noise term for measurement noise. For our determination of $\tau_{c,total}$, we fit the model of Supplementary Equation (12) to measurements of K obtained with different exposure times as detailed in Methods. The definition of x differs a factor $\sqrt{\pi}/2$ from other publications[1,15,16] in which $g_1(\tau) = \exp(-(\tau/\tau_c)^2)$ is used – a form that is incompliant with our Supplementary Equation (8). In biological tissue $\tau_{c,total}$ represents the total decorrelation due to contributions from 'offset' (e.g. muscle movements) and the desired flow dynamics.

IV - Monte Carlo simulations to estimate N

To get a reliable estimate for the number of dynamic scattering events, *N*, we built the geometry in Monte Carlo simulation freeware[17,18], and included the scattering coefficients of the static phantom and dynamic polystyrene spheres for all sizes and volume fractions (see Supplementary table 2), as shown in Supplementary Fig. 3. Photon transport was simulated for one fibre, and the results were rotated 90, 180 and 270 degrees to mimic 1 fibre in each corner. In this set-up the optical absorption is low and can be ignored. The simulated detection area faithfully represented the Microscan detection geometry, and only photons within the imaging NA were included for further analysis. The number of dynamic scattering events are stored per detected photon. We simulated 5000 dynamic photons (thus photons with at least 1 scattering event in the flow tube) detected at the centre of the tube region in the field of view (mimicking the experimental image analysis) and calculated the average number of dynamic scattering events, *N*, per dynamic photon. The relationship between μ_s and *N* could be linearly fitted by $N = 1.2\mu_s d$, ($r^2 = 0.99$) for 15 different simulations with μ_s ranging from 20 - 150 mm^{-1} and *d* the tube diameter of 0.2 mm (see Supplementary Fig. 3b). The factor F = 1.2 [95% upper CI - lower CI: 1.21 - 1.26, CI confidence interval] is likely due to an increased effective optical path length as a result of scattering, photons crossing the tube with a longitudinal component, and photons crossing the tube more than once. For low μ_s (< 20 mm^{-1}) the factor was slightly higher (< 1.9), due to a low number of scattering events in the tube (*N* < 5) and subsequent overestimation of the photon path through the tube. We therefore fixed the factor at 1.2 for all μ_s and this specific *d* of 0.2 mm.

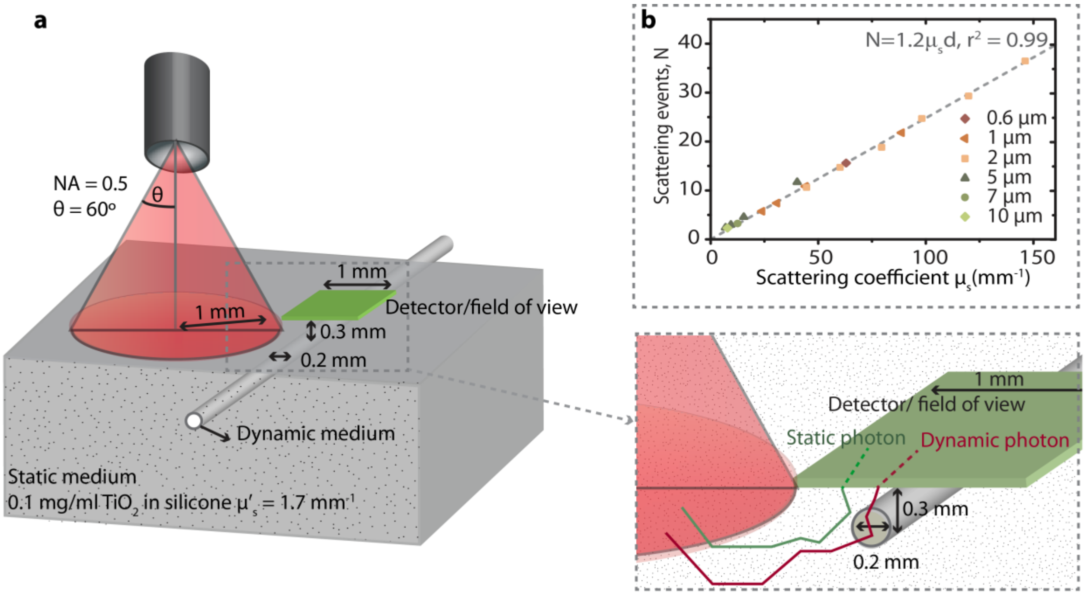

Supplementary Fig. 3 | Monte Carlo geometry and simulation *in vitro*. (a) To mimic the SDF geometry a diverging beam is located at ~1 mm distance from the detector field representing a high NA fibre and photons are launched into the static medium (TiO_2 in silicone). The flow tube with optical properties for polystyrene spheres is placed in the medium (diameter 0.2 ± 0.03 mm; depth 0.3 mm ± 0.03 mm). The simulation results are rotated to represent a fibre in each corner. For each photon detected at the centre of the tube (in the detector plane) the number of dynamic scattering events *N* is counted, and the average *N* per detected photon is plotted versus scattering coefficient in **(b)**, as well as a linear fit to the data (dashed line).

The number of scattering events in the *in vivo* vessels was also estimated using Monte Carlo simulations with the same imaging and detection geometry as shown in Supplementary Fig. 3. To reduce computation time we adopted a different method as compared with the flow phantom: since whole blood and soft tissue have comparable reduced

scattering coefficients[19], we simulated the photon paths in a medium with constant μ'_s = 1.7 mm^{-1} and recorded all coordinates (x,y,z) of each scattering event along the path from illumination fiber to detector element. Next, we placed virtual vessels of different diameters inside the medium and calculated for each detected photon the number of scattering events *N* that had taken place within the vessel volume. Upon inspection, the value of *N* was robust for different locations and depths of the vessel within the field-of-view and focal plane maximal depth (400 μm) and mainly depended on the vessel diameter. In addition, the pixels within 1.5× the diameter of the vessel recorded similar values of *N*. Thus, by placing the virtual vessel at several locations in the medium and obtaining *N* for pixels within a certain distance from the vessel center a statistically valid value for *N* could be found within reasonable computational time. We verified that the results obtained using this approach gave similar results as when a true vessel geometry was simulated. The optical properties of human blood were obtained from a review by Bosschaart et al.[20]. We calculated the optical properties accordingly, taking into account a reduced microcirculatory hematocrit (Hct) as a result of the Fahraeus effect[21] (average microcirculatory Hct = 30%, see Supplementary table 1), for a range of vessel diameters [0.02 - 0.4 mm]. The resulting *N* can be described by $N = (0.92d+0.92)\mu_s d$, with *d* in mm and μ_s = 95 mm^{-1}. For chick embryo whole blood no experimentally validated optical properties were found, therefore, we estimated the optical properties using the known red blood cell volume [22] and hematocrit [23] calculated using Mie scattering and Pervus-Yevick theories[2,4], see Supplementary table 1. Inserting these value into the Monte Carlo geometry resulted in $N = (0.68d+0.92)\mu_s d$, with μ_s = 86 mm^{-1}. To find *N* the *in vivo* vessel diameters can be estimated from conventional SDF images.

To generalize our results, we simulated two other scenarios: 1. *N* for vessels with full hematocrit (Hct = 45%) and 2. *N* for a common LSCI system geometry. In most LSCI systems, the laser light illuminates the tissue at a 45º angle and the detector is placed perpendicularly above the tissue. For all situations similar values for *N* were found, as is plotted in Supplementary Fig. 4 below.

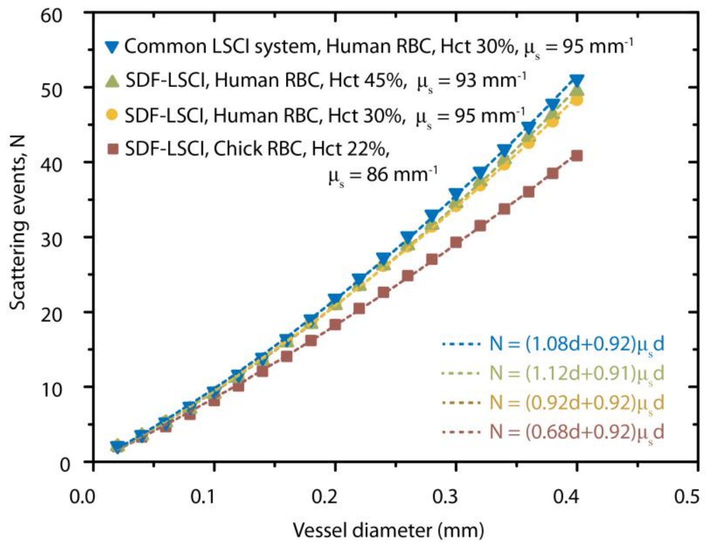

Supplementary Fig. 4 | *In vivo* scattering events from Monte Carlo simulations. The average *N* per detected dynamic photon for a range of vessel diameters was obtained from Monte Carlo simulations using *in vivo* optical properties for human (Hct 30% (yellow circles) and Hct 45% (green triangles)) and chick embryo (Hct 22% (red squares)) blood, see Supplementary table 1. In addition, *N* was simulated for a common LSCI system where the laser beam is directly illuminating the tissue at a 45º angle and the camera is placed perpendicularly above the tissue (blue upside down triangles). Polynomial curve fits are plotted as dashed lines and the fit coefficients are displayed (all r^2 > 0.99).

V - Optical properties of scatterers

Supplementary table 1 and 2 list the optical properties of scatterers used in the *in vivo* and *in vitro* experiments. To estimate the error in theoretical α we varied the size of the polystyrene spheres by ±10% and the refractive index by ±5%, as this variation was reported for the purchased polystyrene spheres (Kisker-Biotech, Germany) [24]. For the red blood cells (RBCs) we varied the size by ±5% and refractive index by ±1%.

Table 1. Optical properties of scatterers at 632.8 nm[a]

	Refractive index particle	Refractive index medium	Scatterer diameter	Vol %	Scattering coefficient	g^b
Human blood, microcirculation	n_{RBC} = 1.3937[c]	n_{plasma} = 1.345[c]	5.6 µm[d]	30%	$\mu_{s,blood}$ = 95 mm^{-1}	0.991
Human blood	n_{RBC} = 1.3937[c]	n_{plasma} = 1.345[c]	5.6 µm[d]	45%	$\mu_{s,blood}$ = 93 mm^{-1}	0.986
Chick blood, microcirculation	n_{RBC} = 1.3937[c]	n_{plasma} = 1.345[c]	7.1 µm[d]	22%[e]	$\mu_{s,blood,chick}$ = 42 mm^{-1}	0.993
TiO_2 in silicone	n_{TIO2} = 2.49[f]	$n_{silicone}$ = 1.4225[f]	~0.2 µm[f]	0.0265% (1 mg/ml)	$\mu_{s,phantom}$ = 4.1 mm^{-1}	0.58
Polystyrene spheres	n_{sphere} = 1.5872	n_{water} = 1.332	see Suppl. table 2	see Suppl. table 2	see Suppl. table 2	see Suppl. table 2

[a] Calculated using Mie and Percus-Yevick theory[25]. [b] Anisotropy (*g*) is the average cosine of the scattering angle. [c] See ref [20]. [d] Calculated for an equivolumetric sphere with the same volume as an human/chick RBC[20,22] [e] See ref. 23 [f] See ref. 26.

Table 2. Optical properties of polystyrene sphere solutions in water at 632.8 nm[a]

Size series			Volume fraction series								
2.5 vol%			1 µm			2 µm			5 µm		
D[b]	μ_s [c]	g[d]	vol%	μ_s [c]	g[d]	vol%	μ_s [c]	g[d]	vol%	μ_s [c]	g[d]
0.6	63	0.848	0.64	24	0.917	1.9	45	0.912	1.1	6.9	0.864
1	89	0.914	0.83	31	0.917	2.5	58	0.911	1.5	9.4	0.864
2	58	0.911	1.2	44	0.916	3.5	80	0.909	2.5	15	0.862
5	15	0.862	2.5	89	0.914	4.4	98	0.908	4.1	25	0.859
7	13	0.918				5.5	120	0.906	6.9	40	0.854
10	7.5	0.899				6.9	147	0.903			

[a]Calculated using Mie and Percus-Yevick theory[25]. [b]D is diameter in µm. [c] µs in mm-1.
[d]Anisotropy (*g*) is the average cosine of the scattering angle.

VI - Multiple scattering scaling factor for chick embryo blood

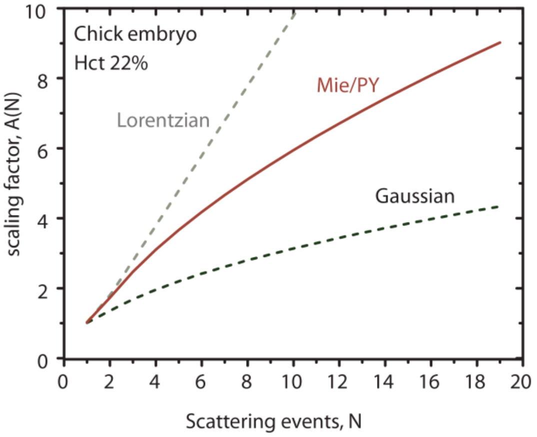

Supplementary Fig. 5 | Multiple scattering scaling factor for α, *A(N)*, for chick embryo blood. $A(N) = \alpha/\alpha_1$, is plotted versus multiple scattering events, N, in chick embryo blood calculated using our model based on Mie-Percus-Yevick scattering (red line). Dashed lines represent $A(N)$, for Lorentzian (grey) and Gaussian (black) models for g_1. All scaling factors are calculated using a normal distribution for the number of scattering events in the vessel $p_N(n)$ for Hct = 22%, with mean N and variance determined by the Percus-Yevick pair correlation function (Supplementary equation 6).

VII - Microcirculatory flow mapping

With conventional-SDF and SDF-LSCI images at multiple exposure times at hand, a quantitative blood flow map can be constructed. We applied the following steps:

1. Raw speckle images were converted to K-images by calculating the ratio of standard deviation and mean intensity at each pixel from a local neighbourhood using a spatiotemporal local region of 7 x 7 (spatial) x 20 (temporal) pixels.
2. For each pixel in the K-image a multi-exposure curve fit is performed (with a priori estimated β_M and ρ as fixed parameters, as described in Methods and Supplementary Section IIId) to estimate $\tau_{c,total}$.
3. To correct for offset decorrelation $\tau_{c,offset}$ is estimated as the average of the lowest 10% of pixels in the $1/\tau_{c,total}$ map and τ_c is calculated per pixel according to[1]:

$$\tau_c = \tau_{c,offset} \cdot \tau_{c,total} \Big/ \left(\tau_{c,offset}{}^2 - \tau_{c,total}{}^2 \right)^{1/2} \tag{13}$$

   An example of such $1/\tau_c$ map (perfusion map) in false colour is shown in Fig. 7b (human, main paper) and Supplementary Fig. 6b (chick embryo).
4. For selected vessels the diameter is estimated from the conventional-SDF images and N is estimated following Supplementary Fig. 4 based on Monte Carlo simulations of our measurement geometry.
5. Selected vessels are manually masked based on conventional SDF images and $1/\tau_c$ is corrected for multiple scattering according to $1/\tau_{c,1} = (1/\tau_c)/A(N)$, see Fig. 2 (human, main paper) and Supplementary Fig. 5 (chick embryo)
6. The resulting map is represented in false colour to visualize the blood flow (Fig. 7c main paper and Supplementary Fig. 7c), using $1/\tau_{c,1} \approx [0.38 \pm 0.04]V$ (human) or $1/\tau_{c,1} \approx [0.20 \pm 0.07]V$ (chick embryo)

This practical guide shows the feasibility of quantitative flowmetry. We identified the following revisions for enhanced performance. Step 3: The offset decorrelation correction would be improved if a site adjacent to each vessel is selected to estimate $\tau_{c,offset}$. Step 4: The vessel diameter can vary along a vessel and at intersections, therefore an improved estimate of N can be obtained by measuring the diameter dynamically along the vessels. Step 5: Vessel masking is done manually for selected vessels, automatic masking will be more complete and efficient.

In Supplementary Movie 2 a video representation of Supplementary Fig. 6 is shown for the chick embryo microcirculation.

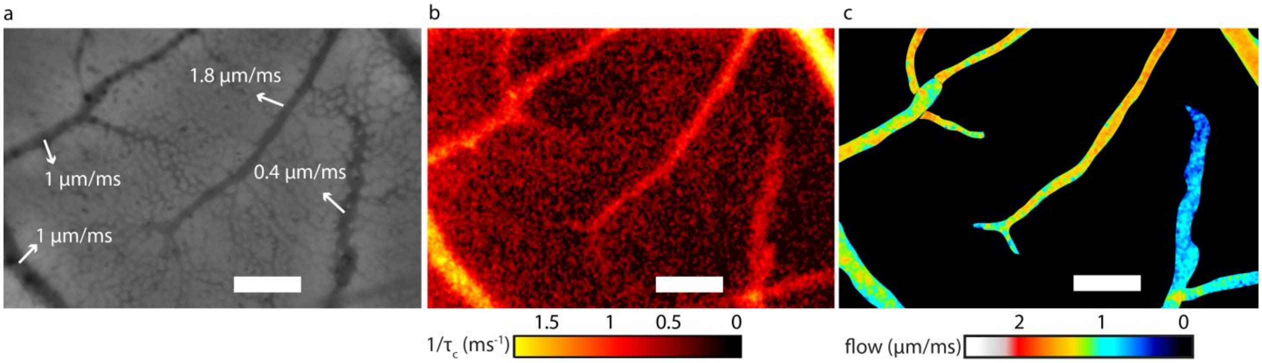

Supplementary Fig. 6 | Blood flow mapping of chick embryo chorioallantoic membrane. (a) Conventional SDF image with contrast due to higher absorption of (green) light by RBCs than by tissue. Flows below 2 mm/s can be measured by RBC tracking. **(b)** $1/\tau_c$ map of the same microcirculation region obtained with multi-exposure SDF-LSCI with contrast due to perfusion dynamics **(c)** Map of LSCI-derived blood flow velocity after correction for $\tau_{c,offset}$ and $A(N)$ (See Supplementary Fig. 5), and masking of selected blood vessel contours. The scale bar is 100 µm.

VIII - Theoretical prediction for α versus scatterer diameter (polystyrene spheres)

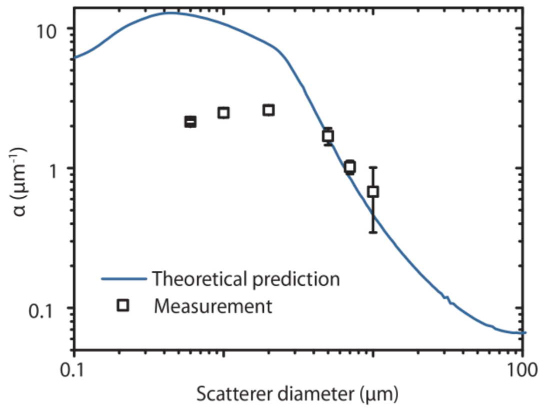

Supplementary Fig. 7 | α versus size. Measurement and theoretical prediction for α *in vitro* versus scatterer diameter in logarithmic scale showing the saturation of α for small diameters. Error bars are 95% CI intervals. Theoretical α is derived using Mie-Percus-Yevick scattering approximations and the number of scattering events N as obtained from Monte Carlo simulations (N =1.2$\mu_s d$) for the *in vitro* flow phantom setup.

IX - *In vivo* determination of α without the offset-correction.

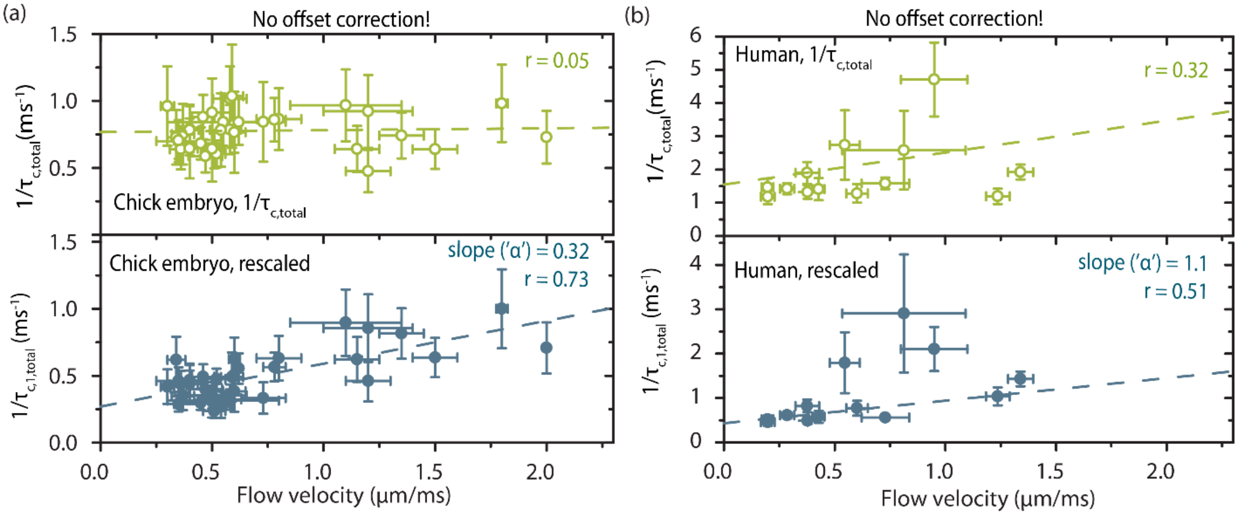

Supplementary Fig. 8 | *In vivo* determination of α without the offset-correction. $1/\tau_{c,total}$ versus V for RBCs *in vivo*, for **(a)** chick embryo and **(b)** human microcirculation. The top panels (green open circles) show $1/\tau_{c,total}$ estimated by a multi-exposure curve fit (Supplementary Section IIId, equation 12), without subsequent offset correction. The bottom panels show $1/\tau_{c,1,total}$ rescaled for the average number of scattering events N, using model based $A(N)$. In both (a) and b) one data point was excluded as an outlier (not shown). Vertical error bars represent 95% CI of the multi-exposure curve fit and horizontal error bars represent the standard deviation in reference flow velocity measurements from conventional SDF images. For the non-offset-corrected data, the slope, or α_1, is 0.32 and 1.1 for chick embryo respectively human RBCs, instead of α_1 is 0.20 and 0.39 (offset corrected, chick embryo respectively human). The offset-corrected α_1 thus approaches the theoretical prediction for α' much better (Theoretical α_1 is 0.27 (chick embryo) respectively 0.38 (human)), especially for the human data. Thus, the data show that for human sublingual microcirculation the offset-correction is essential. It is less important for chick embryo vessels, as they are embedded in a hardly scattering medium (egg-white) and have a low vessel density, resulting in long offset decorrelation times (Supplementary equation 13).

X - Experimental validation of Monte Carlo simulations

The average number of scattering events N in the tube (phantom experiment) was estimated with Monte Carlo simulations (Supplementary Section IV), and yielded: $N = F\mu_s d$, with F = 1.2. We validate this estimation using experimental values of ρ (the fraction of detected photons that have been dynamically scattered). The probability of photons reaching the vessel is denoted c_1. The probability of crossing the vessel with path length l without scattering events follows from Beer's law as $exp(-\mu_s l)$, where μ_s is the scattering coefficient. Normalizing the path length on vessel diameter d using $l=F\cdot d$ we obtain the probability for passing the vessel under the condition of one or more scattering events as $[1-exp(-\mu_s F\cdot d)]$ so that ρ can be estimated as $\rho = c_1 [1-exp(-\mu_s F\cdot d)]$. In Supplementary Fig. 9 ρ is plotted for all *in vitro* measurements. The best fit yielded c_1 = 0.83 and F = 0.95 [95% upper CI - lower CI: 0.81 - 1.1], r^2 = 0.83. Fixing F = 1.2 to match the Monte Carlo estimation resulted in r^2 = 0.74. Both fits are plotted in Supplementary Fig. 9 and resemble the data. This analysis confirms our simulated estimate for N.

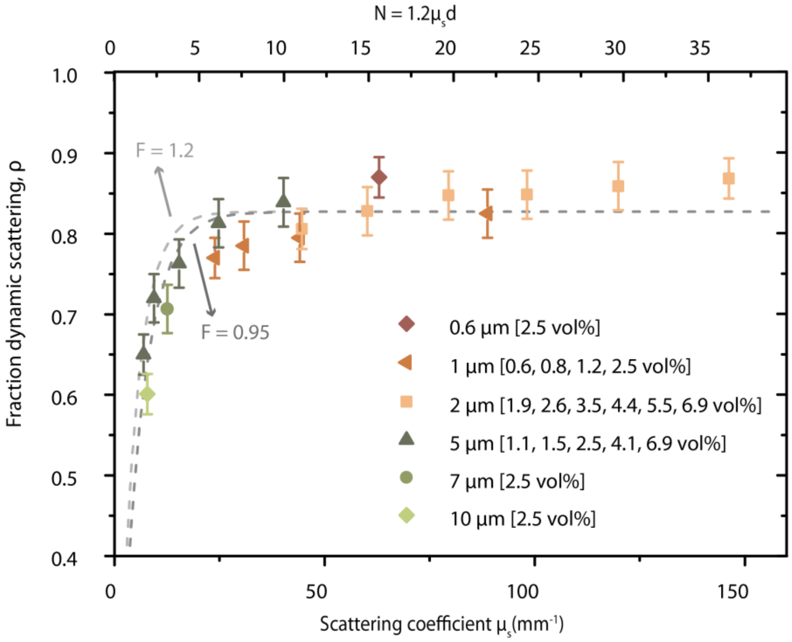

Supplementary Fig. 9 | Fraction dynamically scattered light (ρ) versus scattering properties. The dashed line represents a curve fit of $\rho = c_1 [1-exp(-\mu_s F\cdot d)]$with fit parameters c_1 = 0.83 and F = 0.95 (r^2 = 0.83), or F = 1.2 (r^2 = 0.74)

XI – Influence of Gaussian approximation to *K(T)*

The decorrelation time τ_C is derived from the measurement of spatial speckle contrast *K* vs. integration time *T* by fitting an analytical model to the measurements. The model in Supplementary equation (12) assumes a Gaussian form of the autocorrelation function $g_1(\tau)$ which we justify by the approximately Gaussian form of all distributions in the model:

- The velocity distribution *P(V)* is assumed to Gaussian because of the randomization of the scattering vector $\boldsymbol{q}$ with respect to the flow velocity vector **V**. This assumption is not expected to hold all experimental geometries; e.g. for collimated incident light on a laminar flow profile.
- The phase function $p(\boldsymbol{q})$ is approximately Gaussian for particles with little refractive index difference from their suspended medium. For large particles with high refractive index contrast this assumption has limited accuracy.
- The distribution of the number of scattering events is Poissonian for highly diluted samples. We assume a Gaussian distribution of *n* which will be less valid for small number of scattering events.

We calculated $g_1(\tau)$ and *K(T)* using our model for RBCs, and N = 1, 5 and 50 scattering events, respectively, yielding $\tau_c(1)$=2.66 ms; $\tau_c(5)$=0.78 ms and $\tau_c(50)$=0.18 ms. Supplementary Fig. 10 shows both groups of curves simulated for a flow velocity V = 1 µm/ms. We then calculate $g_1(\tau)$ and *K(T)* for the Gaussian ACF using the respective values for τ_c (Supplementary equation (8)). Subsequent scaling of the time-axes on τ_c causes these latter curves to overlap. For reference, $g_1(\tau)$ and *K(T)* based on the Lorentzian model (appropriate when Brownian motion is considered) are also provided. The three forms of g_1 are listed in the table next to Supplementary Fig. 10 and calculated for multiple scattering using supplementary equation (5) and a normal distribution for $p_N(n)$. The $\langle\ldots\rangle_{\boldsymbol{q}}$ represents integration over the phase function. From the close resemblance of *K(T)* curves using our model of $g_1(\tau)$ and that obtained from a Gaussian form with the same τ_c, we conclude that τ_c can be retrieved adequately using Supplementary equation (12). We note that this result does not imply that $g_{1,single}(\tau)$ is Gaussian nor that the scaling factor based on this assumption should be used ($A(N) \sim \sqrt{N}$, reference curve in Supplementary Fig. 5 and main paper Fig. 2). Rather, the *K(T)* curve at any number of scattering events can be fitted with Supplementary equation (12) to yield τ_c and model based rescaling to $\tau_{c,1}$ follows from the Mie/PY curve in Supplementary Fig. 5 and main paper Fig. 2.

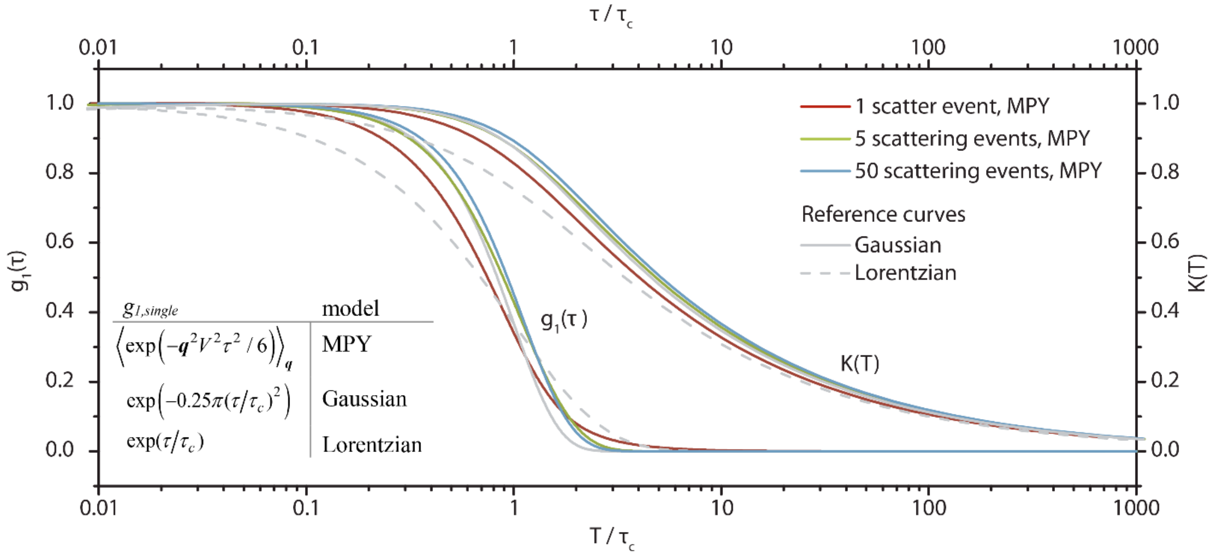

Supplementary Fig. 10 | Theoretical modelling of $g_1(\tau)$ and $K(T)$ in the multiple scattering regime. $g_1(\tau)$ and $K(T)$ are theoretically calculated for the optical and geometrical properties of RBCs, for N = 1 (red), 5 (green) and 50 (blue) scattering events, yielding τ_c = 2.66 ms, τ_c = 0.78 ms and τ_c = 0.18 ms for N = 1, 5, 50 respectively. Gaussian (solid line) and Lorentzian (dashed line) models with the same τ_c are also plotted. A flow velocity of V = 1 µm/ms was used in the calculation. The three forms of $g_1(\tau)$ are listed in the table, where the $\langle\ldots\rangle_{\boldsymbol{q}}$ represents integration over the phase function.

XII - Relation to Diffusing Wave Spectroscopy and Laser Doppler Flowmetry

The model presented in Supplementary Section I is of similar form and origin as the theory for Diffusive Wave Spectroscopy (DWS)[27,28] and for Laser Doppler flowmetry (LDF)[8]. Our model differs in two key areas with both. The first area covers the different approaches in deriving the ACF for single scattering events $g_{1,single}$. Rather than carrying out the integration over the velocity distribution, leading from Supplementary equation (2) to Supplementary equation (3), DWS uses a cumulant expansion for short times τ to bring the ensemble average into the exponential:

$$g_{1,\mathrm{single}}(\tau)=\begin{cases}\left\langle \exp\left(-\boldsymbol{q}^2V^2\tau^2/6\right)\right\rangle_{\boldsymbol{q}} & \text{This work, Supplementary equation (4)}\\ \exp\left(-\left\langle \boldsymbol{q}^2\right\rangle_{\boldsymbol{q}} V^2\tau^2/6\right) & \text{Diffusive Wave Spectroscopy, ref [21]}\end{cases} \tag{14}$$

Where the $\langle\ldots\rangle_{\boldsymbol{q}}$ represents integration over the phase function. For DWS, $\langle \mathbf{q}^2\rangle_{\mathbf{q}}$, evaluates to $2k_0^2(1-g)$ where g is the average cosine of the scattering angle. Then, the scaling factor α' between the inverse of the decorrelation time and flow velocity, $1/\tau_C = \alpha_1 V$ becomes:

$$\alpha_{1,\mathrm{DWS}} = k_0\sqrt{\frac{1}{3}(1-g)} \tag{15}$$

We note that the dependence on particle diameter and volume fraction is included in g. Using Supplementary Table I we find $\alpha'_{\mathrm{DWS}} \approx 0.54$ in the human microcirculation. The LDF framework proceeds to integrate Supplementary equation (14) over $\boldsymbol{q}$, weighted by the form factor, which is equivalent to our weighting with the phase function. However, LDF uses the Rayleigh-Gans (RG) approximation to estimate the angular distribution of scattered light, whereas we use the more generically applicable Mie theory combined with Percus-Yevick equation to account for volume fraction effects. LDF[8] proceeds to approximate the RG phase function by a Gaussian form leading to

$$\alpha_{1,LDF} \approx \frac{1}{1.35r} \tag{16}$$

Where r is the particle radius. Our definition of τ_c in Supplementary equation (8) differs from the definition by Bonner and Nossal[8] who use $\tau_{½}$, which is related to τ_c by $\tau_{1/2} = \tau_c\sqrt{4\ln 2/\pi}$. For comparisons reasons we adhere to our definition which explains the difference of the factor 1.35 with the factor 1.27 that is often cited in literature. For human blood we find $\alpha_{1\mathrm{LDF}} \approx 0.26$.

The second key difference concerns how multiple scattering is treated. Our summation over the number of scattering events n is replaced by integration over the path length distribution $p(l)$. The two quantities are related through $l=n/\mu_s$:

$$g_1(\tau)=\begin{cases}\sum_n p(n)\left\langle \exp\left(-n\boldsymbol{q}^2V^2\tau^2/6\right)\right\rangle_{\boldsymbol{q}} & \text{This work, Supplementary equation (5)}\\ \int p(l)\exp\left(-2k_0^2(1-g)V^2\tau^2 l/6\right) & \text{Diffusive Wave Spectroscopy, ref [21]}\end{cases} \tag{17}$$

Expressions for $p(l)$ can then be derived from e.g. the radiative transport equation[28], or, similar to our approach, approximated from Monte Carlo simulations[29]. LDF assumes the Poissonian form of $p_N(n)$ to carry out the summation, thereby essentially limiting the range of validity to low volume fractions. Contrary to the coherent techniques discussed here, low coherence interferometry allows control over $p(l)$, either coercing the experiment to single scattering[30] or low-order scattering[31]. The latter work proposes an empirical correction to the assumed Poissonian form of $p(l)$. Part of the correction is related to instrumental factors, part is attributed to volume fraction-dependent effects on the optical properties (correctly so in our opinion for the reasons given in Supplementary section IIIc). The choice of $p_N(n)$ becomes of lesser influence as the mean number of scattering events N increases, because all forms of $p_N(n)$ eventually become Gaussian as per the Central Limit Theorem.

XIII – Calculation of tissue perfusion using SDF-LSCI

As described in the introduction, blood flow is the blood volume per unit time in the vasculature, and perfusion is the blood volume per volume tissue, per unit time. Therefore with the vessel diameters obtained from conventional SDF images the flow can simply be calculated as $\pi r^2 V$.

Tissue perfusion can be calculate by the ratio of the fractional volume over the mean transit time of blood. In our case this can be obtained by the ratio of the total vessel volume over the field-of-view x depth-of-field, divided by the transit time, thus vessel length / velocity:

$$\text{Tissue Perfusion} = \frac{\text{fractional volume}}{\text{mean transit time}} = \frac{\dfrac{\sum_i \pi r_i^2 l_i}{FOV * DOF}}{\sum_i \dfrac{l_i}{V_i}} = \frac{\sum_i \pi r_i^2 V_i}{FOV * DOF} \qquad (18)$$

where FOV, field-of-view, DOF, depth-of-focus, r_i,l_i and V_i the radius, length and velocity of each vessel i in the image. Thus, in order to quantitatively measure tissue perfusion using laser speckle flowmetry, a quantitative relationship between τ_C and V needs to be established, as done in this Article using vessel diameters and their relationship to dynamic scattering events N.

Supplementary Movie legends

Supplementary Movie 1 (human) represents the analysis steps taken to generate a quantitative blood flow velocity map, as outlined in Supplementary Section VII. The top-left panel shows conventional SDF videos of the human microcirculation and the bottom-left panel shows speckle-contrast images of the same microcirculation area, calculated from raw laser speckle contrast images taken at different exposure times using Supplementary equation (10). The bottom-right image shows the decorrelation map, off-set corrected, generated using Supplementary equations (12) and (13). Estimation of vessel diameter and N is done using the conventional SDF images to apply $1/\tau_{c,1} = (1/\tau_c)/A(N)$, and subsequently, using $1/\tau_{c,1} \approx [0.38 \pm 0.04]V$, the blood flow velocity map is generated as shown in the top-right of the movie.

Supplementary Movie 2 (chick embryo) represents the same steps as Supplementary Movie 1, for an area of the vascularised chorioallantoic membrane of the chick embryo grown *ex ovo*, and using $1/\tau_{c,1} \approx [0.20 \pm 0.07]V$ in the final step.

References

1 Nadort, A., Woolthuis, R. G., van Leeuwen, T. G. & Faber, D. J. Quantitative laser speckle flowmetry of the in vivo microcirculation using sidestream dark field microscopy. *Biomed. Opt. Express* **4**, 2347-2361 (2013).

2 Hulst, H. C. *Light scattering by small particles*. (Courier Dover Publications, 1957).

3 Percus, J. K. & Yevick, G. J. Analysis of classical statistical mechanics by means of collective coordinates. *Phys. Rev.* **110**, 1-13 (1958).

4 Wertheim, M. Exact solution of the Percus-Yevick integral equation for hard spheres. *Phys. Rev. Lett.* **10**, 321-323 (1963).

5 Tsang, L., Kong, J. A., Ding, K.-H. & Ao, C. O. *Scattering of electromagnetic waves, Numerical simulations*. Vol. 25 (John Wiley & Sons, 2004).

6 Berne, B. J. & Pecora, R. *Dynamic light scattering: with applications to chemistry, biology, and physics*. (Courier Dover Publications, 2000).

7 Boas, D. & Yodh, A. Spatially varying dynamical properties of turbid media probed with diffusing temporal light correlation. *JOSA A* **14**, 192-215 (1997).

8 Bonner, R. & Nossal, R. Model for laser Doppler measurements of blood flow in tissue. *Appl. Opt.* **20**, 2097-2107 (1981).

9 Goodman, J. W. *Speckle phenomena in optics: theory and applications*. 59-139 (Roberts and Company Publishers, 2007).

10 Hansen, J.-P. & McDonald, I. R. *Theory of Simple Liquids*. (Elsevier, 1990).

11 Tsang, L., Kong, J. A. & Ding, K.-H. *Scattering of Electromagnetic Waves, Theories and Applications*. Vol. 27 (John Wiley & Sons, 2004).

12 Boas, D. A. & Dunn, A. K. Laser speckle contrast imaging in biomedical optics. *J. Biomed. Opt.* **15**, 011109 (2010).

13 Parthasarathy, A. B., Tom, W. J., Gopal, A., Zhang, X. & Dunn, A. K. Robust flow measurement with multi-exposure speckle imaging. *Opt. Express* **16**, 1975-1989 (2008).

14 Zakharov, P., Völker, A., Buck, A., Weber, B. & Scheffold, F. Quantitative modeling of laser speckle imaging. *Opt. Lett.* **31**, 3465-3467 (2006).

15 Duncan, D. D. & Kirkpatrick, S. J. Can laser speckle flowmetry be made a quantitative tool? *JOSA A* **25**, 2088-2094 (2008).

16 Parthasarathy, A. B., Kazmi, S. & Dunn, A. K. Quantitative imaging of ischemic stroke through thinned skull in mice with Multi Exposure Speckle Imaging. *Biomed. Opt. Express* **1**, 246-259 (2010).

17 de Mul, F. F. M. in *Handbook of Coherent Domain Optical Methods, Biomedical Diagnostics, Environment and Material Science* (ed Valery V. Tuchin) Ch. 12, 465-533 (Kluwer Publishers, 2004).

18 de Mul, F. F. M. *et al.* Laser Doppler velocimetry and Monte Carlo simulations on models for blood perfusion in tissue. *Appl. Opt.* **34**, 6595-6611 (1995).

19 Jacques, S. L. Optical properties of biological tissues: a review. *Physics in medicine and biology* **58**, R37 (2013).

20 Bosschaart, N., Edelman, G. J., Aalders, M. C., van Leeuwen, T. G. & Faber, D. J. A literature review and novel theoretical approach on the optical properties of whole blood. *Lasers Med. Sci.* **29**, 453-479 (2014).

21 Pries, A. R., Secomb, T. W., Gaehtgens, P. & Gross, J. Blood flow in microvascular networks. Experiments and simulation. *Circul. Res.* **67**, 826-834 (1990).

22 Chan, L. Changes in the composition of plasma membrane proteins during differentiation of embryonic chick erythroid cell. *Proc. Natl. Acad. Sci. U.S.A.* **74**, 1062-1066 (1977).

23 Johnston, P. M. Hematocrit Values for the Chick Embryo at Various Ages. *Am. J. Physiol.* **180**, 361-362 (1955).

24 Nicolet, A. & Meli, F. Report on the needs, specifications and commercial sources of microvesicle reference materials. (EMRP JRP HLT02 MetVes, 2012).

25 Nguyen, V. D., Faber, D., van der Pol, E., van Leeuwen, T. & Kalkman, J. Dependent and multiple scattering in transmission and backscattering optical coherence tomography. *Opt. Express* **21**, 29145-29156 (2013).

26 de Bruin, D. M. *et al.* Optical phantoms of varying geometry based on thin building blocks with controlled optical properties. *J. Biomed. Opt.* **15**, 025001 (2010).

27 Pine, D., Weitz, D., Chaikin, P. & Herbolzheimer, E. Diffusing wave spectroscopy. *Phys. Rev. Lett.* **60**, 1134-1137 (1988).

28 Weitz, D., Zhu, J., Durian, D., Gang, H. & Pine, D. Diffusing-wave spectroscopy: The technique and some applications. *Phys. Scripta* **T49**, 610-621 (1993).
29 Rice, T. B. *et al.* Quantitative, depth-resolved determination of particle motion using multi-exposure, spatial frequency domain laser speckle imaging. *Biomed. Opt. Express* **4**, 2880-2892 (2013).
30 Kalkman, J., Sprik, R. & van Leeuwen, T. Path-length-resolved diffusive particle dynamics in spectral-domain optical coherence tomography. *Phys. Rev. Lett.* **105**, 198302 (2010).
31 Wax, A., Yang, C., Dasari, R. R. & Feld, M. S. Path-length-resolved dynamic light scattering: modeling the transition from single to diffusive scattering. *Appl. Opt.* **40**, 4222-4227 (2001).
